# Weighted Vest Use or Resistance Exercise to Offset Weight Loss–Associated Bone Loss in Older Adults: A Randomized Clinical Trial

**DOI:** 10.1001/jamanetworkopen.2025.16772

**Published:** 2025-06-20

**Authors:** Kristen M. Beavers, S. Delanie Lynch, Jason Fanning, Marjorie Howard, Erica Lawrence, Leon Lenchik, Sue A. Shapses, Ashley A. Weaver, Sarah J. Wherry, Zeke Zamora, Barbara J. Nicklas, Daniel P. Beavers

**Affiliations:** 1 Department of Internal Medicine, Section of Gerontology and Geriatric Medicine, Wake Forest University School of Medicine, Winston-Salem, North Carolina; 2 Department of Health and Exercise Science, Wake Forest University, Winston-Salem, North Carolina; 3 Department of Biomedical Engineering, Wake Forest University School of Medicine, Winston-Salem, North Carolina; 4 Department of Biostatistics and Data Science, Wake Forest University School of Medicine, Winston-Salem, North Carolina; 5 Department of Radiology, Wake Forest University School of Medicine, Winston-Salem, North Carolina; 6 Department of Medicine, Rutgers-RWJ Medical School, New Brunswick, New Jersey; 7 Division of Geriatric Medicine, University of Colorado Anschutz Medical Campus, Aurora; 8 Eastern Colorado Geriatric Research, Education, and Clinical Center (GRECC), Aurora; 9 Department of Statistical Sciences, Wake Forest University, Winston-Salem, North Carolina

## Abstract

**Importance:**

Weight loss (WL) in older adults is associated with bone loss, increasing the risk of fracture. Because skeletal tissue is responsive to mechanical stress, replacing lost weight externally may be an innovative way to minimize WL-associated bone loss in this population.

**Objective:**

To examine the effect of 12 months of weighted vest use during WL on indicators of bone health compared with WL alone and WL plus resistance training (RT).

**Design, Setting, and Participants:**

This single-blind, 12-month randomized clinical trial of older adults living with obesity was conducted at an academic medical center from September 1, 2019, to April 30, 2024.

**Interventions:**

WL (caloric restriction targeting 10% WL with adequate calcium, vitamin D, and protein), WL plus weighted vest (WL+VEST; 8 h/d, weight replacement titrated up to 10% total WL), or WL plus progressive RT (WL+RT; supervised 3 sessions weekly).

**Main Outcomes and Measures:**

Main outcomes included 12-month change in computed tomography–acquired trabecular volumetric bone mineral density (vBMD) and dual-energy X-ray absorptiometry–acquired areal bone mineral density (aBMD) of the total hip. Secondary outcomes included change in additional computed tomography– and dual-energy X-ray absorptiometry–acquired measures of musculoskeletal health and bone turnover biomarkers.

**Results:**

A total of 150 older (mean [SD] age, 66.4 [4.6] years) adults (112 [74.7%] women) living with obesity (mean [SD] body mass index, 33.6 [3.3]) were randomized (50 to WL, 50 to WL+VEST, and 50 to WL+RT), with 133 (88.7%) completing the trial. Similar significant WL, ranging from 9.0% to 11.2%, was achieved in all groups. During 12 months, mean (SD) self-reported weighted vest wear time was 7.1 (1.5) h/d, with 78.0% (29.9%) of lost weight replaced in the vest; participants randomized to the WL+RT group attended a mean (SD) of 71.4% (19.1%) of sessions. A significant decrease in total hip trabecular vBMD was observed at 12 months in all treatment groups (ranging from −1.2% to −1.9%), with no difference between the WL+VEST and WL groups (estimated treatment difference, +0.91 mg/cm^3^; 97.5% CI, −0.27 to 2.09 mg/cm^3^; *P* = .13) and noninferiority of WL+VEST compared with WL+RT (estimated treatment difference, +0.29 mg/cm^3^; 98.75% lower bound, −1.05 mg/cm^3^). Similar effects were observed for total hip aBMD.

**Conclusions and Relevance:**

In this 12-month randomized clinical trial, neither weighted vest use nor progressive RT was able to mitigate WL-associated bone loss at the hip in older adults living with obesity. This study highlights the need for alternative or adjunctive strategies to prevent bone loss in older adults experiencing WL because exercise may be insufficient on its own.

**Trial Registration:**

ClinicalTrials.gov Identifier: NCT04076618

## Key Points

**Question:**

Can weighted vest use mitigate bone loss that occurs when older adults lose weight?

**Findings:**

In this randomized clinical trial of 150 older adults living with obesity who lost approximately 10% of their body weight during a 12-month period, daily weighted vest use did not prevent weight loss–associated bone loss at the hip. The effect of weighted vest use on skeletal health outcomes was largely similar to traditional resistance exercise training.

**Meaning:**

In this trial, bone loss at the hip accompanied dietary weight loss in older adults, the magnitude of which was not affected by weighted vest use or resistance exercise training.

## Teaser

This randomized clinical trial of older adults with obesity examined whether weighted vest use added to weight loss better preserves bone health compared with weight loss alone or weight loss plus resistance training.

## Introduction

The prevalence of obesity and its detrimental health effects is increasing rapidly among older adults.^1,2^ Medical complications associated with excess fat mass indicate a need to treat obesity,^3^ yet recommending weight loss (WL) via caloric restriction is controversial in this age group.^4,5^ Reluctance stems, at least in part, from loss of bone mineral density (BMD) known to accompany WL^6^ and potential exacerbation of age-related risk of osteoporotic fracture, a leading cause of injury in older adults that substantially compromises both quality and expectancy of life.^7,8^

Skeletal tissue is highly responsive to mechanical stress^9^; thus, WL-associated decreases in loading likely contribute to BMD loss.^10,11^ The addition of exercise training, particularly resistance training (RT) designed to enhance gravitational and/or muscle loading, modestly attenuates the amount of bone lost compared with WL alone; however, RT is unable to fully prevent musculoskeletal tissue loss.^12,13,14^ Intervention effectiveness also hinges substantially on exercise adherence. Although intuitive, this observation may be especially important for older adults who are less likely to perform the volume and intensity of exercise necessary to preserve bone during WL.^15^ In addition, conventional RT often requires expensive equipment and on-site participation, as well as safety supervision by trained staff for older adults, limiting its scalability as an intervention strategy.

Treating WL-associated decreases in loading by replacing lost weight externally represents another countermeasure strategy, with demonstrated osteoprotective effects of weighted vest use.^16,17,18,19,20,21,22,23,24^ Intriguingly, pilot data from our group suggest weighted vest use is both feasible and potentially effective in mitigating WL-associated hip areal BMD (aBMD) loss in older adults by increasing bone formation.^25^ The Incorporating Nutrition, Vests, Education, and Strength Training (INVEST) in Bone Health randomized clinical trial (RCT) was conducted to definitively test our hypothesis that weighted vest use added to WL will better preserve bone health compared with WL alone and similarly to WL plus a structured RT intervention among older adults living with overweight or obesity.

## Methods

### Study Design and Oversight

The 12-month INVEST in Bone Health trial was conducted from September 1, 2019, through April 30, 2024, at Wake Forest University, Winston-Salem, North Carolina. Full details of the study design and methods have been previously published,^26^ with a visual schematic of the timeline and outcomes related to the primary outcome study (eFigure in Supplement 1). The study protocol can be found in Supplement 2. The study was approved by the institutional review board of Wake Forest University School of Medicine, and all participants provided written informed consent. The trial was monitored by an independent data and safety monitoring board, and results reporting adheres to the Consolidated Standards of Reporting Trials (CONSORT) reporting guideline.^27^

### Participants

In this intent-to-treat RCT study, participants were recruited through advertisements and underwent a comprehensive screening process, detailed elsewhere.^26^ Briefly, persons were eligible for inclusion if they were aged 60 to 85 years, had a body mass index (BMI; calculated as weight in kilograms divided by height in meters squared) of 30.0 to 40.0 or 27.0 to less than 30.0 plus 1 obesity-related risk factor or clinical comorbidity, had a stable body weight (no WL >5% in past 6 months), and were willing or able to participate in all study procedures and assessments. Those with severe cardiometabolic disease (eg, recent myocardial infarction, unstable angina, uncontrolled hypertension, or diabetes) or musculoskeletal (eg, osteoporosis, severe arthritis, or back pain) impairments that precluded weighted vest use or exercise training, cognitive impairment, or who used prescription osteoporosis or WL medications within the past year were excluded.

### Interventions

From July 1, 2020, to February 29, 2023, 150 participants were randomized (n = 50 per group), using random permuted blocks of 3, 6, 9, and 12 and stratified by gender, to 1 of 3 groups: WL alone, WL plus weighted vest (WL+VEST), or WL plus structured RT (WL+RT). The WL intervention was designed to elicit a 10% loss of initial body mass during the 12-month period and included use of a nutritionally complete, partial meal replacement program (Medifast Inc) along with weekly (first 6 months) or biweekly (second 6 months) group nutrition education classes, which were structured around the group dynamics literature, social cognitive theory, and strategies that optimize self-regulation.^28^ In addition to the WL intervention, participants randomized to the WL+VEST group were also asked to wear a weighted vest 8 h/d during their most active part of the day. Initially, the vest was unloaded (ie, 1-lb vest only), and vest weight was adjusted weekly (using 1/8-lb blocks) to match the total amount of WL experienced by the participant (up to 10% of baseline weight). Participants were asked to keep a daily log to record the time worn, vest weight, and any complications or comments related to the vest use. Participants randomized to the WL+RT group underwent a supervised, progressive RT intervention occurring on 3 nonconsecutive days per week, with a training goal for participants to complete 3 sets of 10 to 12 repetitions for 8 different upper and lower body exercises at 70% to 75% 1 repetition maximum.

### Study Outcomes

The primary study outcome was 12-month change in quantitative computed tomography (CT)–acquired total hip trabecular volumetric bone mineral density (vBMD). Change in total hip aBMD collected via dual-energy x-ray absorptiometry (DXA) was a prespecified secondary outcome. Other secondary measures included additional CT- and DXA-acquired skeletal health measures at the hip, lumbar spine, and distal radius as well as DXA-acquired total body composition. Published short-term precision estimates of BMD measurement by quantitative CT have been calculated as coefficient of variation for vBMD at the lumbar spine (0.80%)^29^ and aBMD at the total hip (0.82%) and femoral neck (0.69%)^30^ using a nominal aBMD of 1.0 g/cm^2^ at the hip. This compares favorably with DXA aBMD measurements, for which published precision estimates are similar for the lumbar spine (1.10%),^31^ total hip (0.65%), and femoral neck (1.66%).^32^ Clinically relevant biomarkers of bone resorption (C-terminal telopeptide) and formation (procollagen 1 intact *N*-terminal propeptide [P1NP]),^33^ self-reported moderate to vigorous physical activity (kilocalories per week) via the Community Healthy Activities Model Program for Seniors (CHAMPS) questionnaire,^34^ and knee extensor strength measured via isokinetic dynamometry were included for relevance as ad hoc outcome measures. All baseline outcome assessments were repeated at 6 and 12 months by trained and blinded assessors, and all assessment methods are detailed in the design article.^26^

### Covariate Assessments

Self-reported demographic (ie, age, gender, race, ethnicity, and educational level) and health (ie, presence of select comorbidities) information were assessed at baseline via survey forms by the study coordinator (E.L.). Preexisting low bone mass was defined as a DXA T score between −1.1 and −2.4 at any regional skeletal site.

### Statistical Analysis

The trial was designed with 50 participants per group to test both hypotheses of the primary aim, with more than 85% power to detect a minimum 2.5% difference in 12-month change in total hip trabecular vBMD between the WL+VEST vs WL group, using a 2-tailed *t* test at a 2-sided *P* = .025 level of significance and greater than 95% power to establish whether the WL+VEST group would experience a noninferior 12-month change in total hip trabecular compared with the WL+RT group using a −4% noninferiority bound based on a 1-sided 98.75% lower confidence bound. Baseline descriptive statistics were summarized overall and by intervention group. The primary aim for comparisons of changes in total hip trabecular vBMD was tested using a mixed-model fit using the change in total hip trabecular vBMD vs the treatment effect indicator for each of the 3 groups, adjusted for visit (6 or 12 months), visit × treatment interaction, gender, and baseline value. A contrast statement tested change in total hip trabecular vBMD at 12 months in the WL vs WL+VEST group, and a statistically significant difference was established at a 2-sided *P* < .025. Next, the noninferiority of WL+VEST compared with WL+RT was determined based on whether the lower bound of the 1-sided 98.75% CI for the estimated 12-month treatment effect of WL+VEST vs WL+RT overlapped the prespecified −4.0% noninferiority boundary for change in total hip trabecular vBMD.^35^ Analyses were repeated for DXA-acquired, 12-month total hip aBMD treatment effects. Comparisons were performed for both superiority of WL+VEST vs WL (α = .025) as well as noninferiority of WL+VEST vs WL+RT (α = .0125) based on a −2.13% noninferiority margin^36^ using the methods described above.

Analyses for all secondary outcomes at 6 and 12 months mirror the model used in the primary aim, and statistical comparisons focused on overall group mean differences. Treatment effects for changes in outcome variables were compared using a mixed-model fit with treatment group, visit, and treatment × visit interaction, adjusted for gender and baseline values of the outcome. Biomarkers were modeled using log transformation to account for skewness. Tests were performed using contrast statements at 6 and 12 months and used the 2-*df*, 2-sided *P* < .05 to indicate statistical significance. Significant comparisons for secondary outcomes used *P* < .0167 for pairwise tests.

## Results

### Participant Characteristics

**Figure 1.**
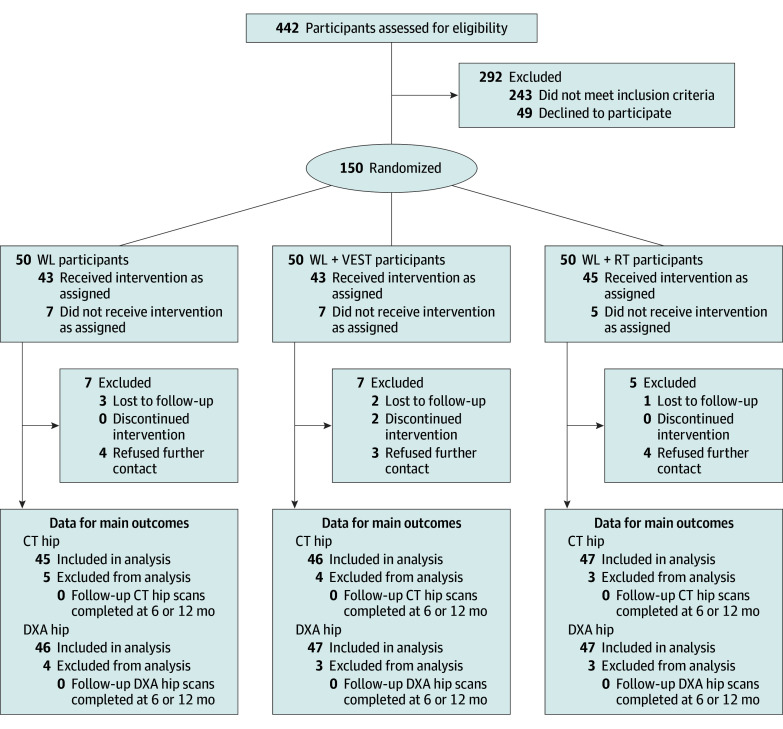
Participant Flow Through the Incorporating Nutrition, Vests, Education, and Strength Training (INVEST) in Bone Health Trial CT indicates computed tomography; DXA, dual-energy X-ray absorptiometry; WL, weight loss; WL+RT, weight loss plus resistance training; WL+VEST, weight loss plus weighted vest.

A total of 150 volunteers were recruited and randomized (50 per group), with 133 (88.7%) completing the study (Figure 1). Baseline characteristics are presented in Table 1. Mean (SD) age was 66.4 (4.6) years; 112 (74.7%) were women and 38 (25.3%) were men; 43 (28.7%) were African American or Black, 3 (2.0%) were Hispanic or Latino, 100 (66.7%) were White, and 4 (2.7%) were other race (multiracial or any other race); and 122 (81.3%) reported a postsecondary educational level. The mean (SD) BMI was 33.6 (3.3), and 73 (48.7%) presented with preexisting low bone mass (regional T score between −1.1 and −2.4). Self-reported moderate to vigorous physical activity (mean [SD], 672.8 [1110.4] kcal/wk) indicated a moderately active sample at baseline. No differences in descriptive and clinical characteristics were noted between completers and noncompleters at baseline (eTable 1 in Supplement 1).

**Table 1.** Baseline Demographic and Clinical Characteristics

Characteristic	No. (%) of participants			
	Overall (N = 150)	WL (n = 50)	WL+VEST (n = 50)	WL+RT (n = 50)
Age, mean (SD), y	66.4 (4.6)	65.9 (4.2)	66.9 (4.5)	66.4 (5.1)
Gender				
Male	38 (25.3)	13 (26)	13 (26)	12 (24)
Female	112 (74.7)	37 (74)	37 (74)	38 (76)
Race and ethnicity				
African American or Black	43 (28.7)	12 (24)	16 (32)	15 (30)
Asian	0	0	0	0
Hawaiian or Other Pacific Islander		0	0	0
Hispanic or Latino	3 (2)	1 (2)	1 (2)	1 (2)
Native American or Alaska Native	0			0
White	100 (66.7)	34 (68)	32 (64)	34 (68)
Other^a^	4 (2.7)	3 (6)	1 (2)	0
Body mass, mean (SD), kg	92.9 (12.8)	92.3 (14.3)	93.6 (12.3)	92.7 (11.9)
Height, mean (SD), cm	166.2 (8.4)	166 (8.8)	166.5 (8.5)	166 (8)
BMI, mean (SD)	33.6 (3.3)	33.4 (3.3)	33.8 (3.7)	33.6 (3)
Educational level				
High school or equivalent (grades 9-12)	28 (18.7)	8 (16)	13 (26)	7 (14)
College (grades 13-16)	81 (54)	31 (62)	23 (46)	27 (54)
Postgraduate	41 (27.3)	11 (22)	14 (28)	16 (32)
Comorbidities				
Diabetes	12 (8)	6 (12)	5 (10)	1 (2)
CVD	16 (10.7)	6 (12)	4 (8)	6 (12)
Arthritis or joint pain	104 (69.3)	36 (72)	39 (78)	29 (58)
Preexisting low bone mass^b^	73 (48.7)	26 (52)	24 (48)	23 (46)
Self-reported moderate-intensity physical activity, kcal/wk				
Mean (SD)	672.8 (1110.4)	613.9 (1239.6)	690.7 (1135.5)	713.9 (958)
Median (IQR)	204.6 (0-791.4)	0 (0-680.6)	196.7 (0-962.1)	412.9 (0-955)
Knee extensor strength, mean (SD), N · m	118.2 (32.3)	113.5 (35.5)	119.3 (24.6)	121.7 (35.2)
CT data, mean (SD)				
Total hip trabecular vBMD, mg/cm^3^	131.9 (21.1)	129.9 (20.4)	131.1 (21.7)	134.7 (21.3)
Total hip cortical vBMD, mg/cm^3^	701.7 (27.2)	699.4 (29.1)	705.1 (24)	700.7 (28.5)
Femoral neck trabecular vBMD, mg/cm^3^	127.4 (24.8)	126.7 (21.1)	125.7 (29.5)	129.9 (23.6)
Femoral neck cortical vBMD, mg/cm^3^	694.7 (35.5)	696.4 (33.2)	700.7 (34.4)	687.1 (37.9)
L1-L4 mean trabecular vBMD, mg/cm^3^	120.8 (36.6)	118.3 (37.1)	119.9 (29.1)	124.2 (43)
DXA data, mean (SD)				
Total hip aBMD, mg/cm^2^	1024.7 (139.5)	1013.2 (139.6)	1026.4 (130.2)	1034.6 (149.9)
Femoral neck aBMD, mg/cm^2^	957.8 (140.5)	958.5 (137.3)	948.7 (147.7)	966.3 (138.6)
Distal radius aBMD, mg/cm^2^	883.2 (110.3)	873.3 (110.1)	899.3 (95.3)	877 (124)
Lumbar spine aBMD, mg/cm^2^	1278.1 (215.5)	1234.4 (180.2)	1287.4 (185.6)	1312.4 (266.7)
Trabecular bone score	1.4 (0.1)	1.4 (0.1)	1.4 (0.1)	1.4 (0.1)
Total body fat mass, kg	40.8 (7.1)	40.9 (7)	40.9 (7.8)	40.7 (6.7)
Total body lean mass, kg	48.3 (9.1)	47.6 (9.9)	48.7 (8.8)	48.8 (8.7)
Appendicular lean mass, kg	22.8 (5)	22.4 (5.5)	22.9 (4.8)	23.2 (4.6)
Blood-based biomarkers, mean (SD)				
CTX, μg/L	0.21 (0.14)	0.21 (0.10)	0.20 (0.14)	0.24 (0.15)
P1NP, μg/L	35.0 (15.7)	36.5 (14.2)	31.6 (13.3)	37.0 (18.9)

Abbreviations: aBMD, areal bone mineral density; BMI, body mass index (calculated as weight in kilograms divided by height in meters squared); CT, computed tomography; CTX, C-terminal telopeptide; CVD, cardiovascular disease; DXA, dual-energy X-ray absorptiometry; P1NP, procollagen 1 intact *N*-terminal propeptide; vBMD, volumetric bone mineral density; WL, weight loss; WL+RT, weight loss plus resistance training; WL+VEST, weight loss plus weighted vest.

^a^ Participants self-reported race and ethnicity. Four participants reported that they considered their race to be multiracial or other, and no further information was collected.

^b^ T score on hip, spine, forearm radius, or femoral neck DXA scan of −1 to −2.49.

### Intervention Process Measures

Significant and similar WL, ranging from 9.0% to 11.2%, was achieved in all groups, with most weight lost by 6 months (WL: change, −8.94 kg; 95% CI, −10.58 to −7.31 kg; WL+VEST: change, −8.68 kg; 95% CI, −10.30 to −7.05 kg; WL+RT: change, −9.61 kg; 95% CI, −11.26 to −7.96 kg) and maintained through 12 months (WL: change, −8.91 kg; 95% CI, −10.77 to −7.04 kg; WL+VEST: change, −8.36 kg; 95% CI, −10.21 to −6.50 kg; WL+RT: change, −10.44 kg; 95% CI, −12.31 to −8.56). In the WL+VEST group, mean (SD) weighted vest wear time during 12 months was 7.1 (1.5) h/d, with a mean (SD) of 78.0% (29.9%) of lost weight replaced in the vest. Overall, 26 of 37 participants (70.3%) with satisfaction survey data assigned to wear the weighted vest were satisfied or highly satisfied with the intervention, with 15 of 37 (40.5%) stating that they would be willing to wear the vest if they were not in the study. Participants randomized to the WL+RT group attended a mean (SD) of 71.4% (19.1%) of classes, with a mean (SD) of 2.1 (0.6) sessions attended per week during 12 months.

### Adverse Events

eTables 2 to 4 in Supplement 1 summarize adverse event (AE) data for the trial. Briefly, a total of 193 AEs occurred after the start of the intervention, with most being classified as other followed by musculoskeletal. As expected, more musculoskeletal events were reported in the WL+VEST (n = 17) and WL+RT (n = 19) groups compared with the WL alone group (n = 6). Of 6 serious AEs reported, none were related.

### Total Hip BMD and Other Musculoskeletal Measures

**Figure 2.**
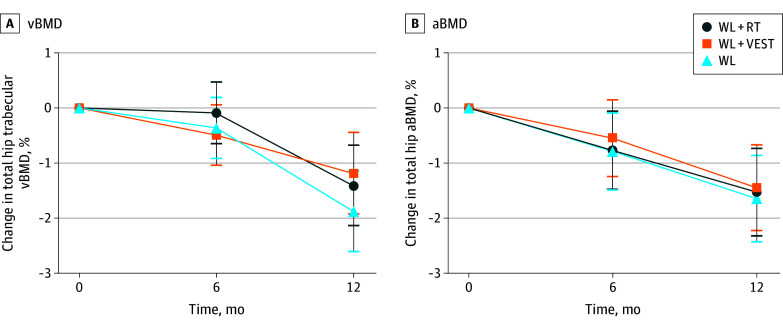
Primary and Prespecified Secondary Outcomes by Treatment Group and Time Data are mean percent change, with error bars representing 95% CIs. aBMD, areal bone mineral density; vBMD, volumetric bone mineral density; WL, weight loss; WL+RT, weight loss plus resistance training; WL+VEST, weight loss plus weighted vest.

Table 2 presents primary (CT-acquired total hip trabecular vBMD) and prespecified secondary (DXA-acquired total hip aBMD) outcomes by treatment group. By 12 months, all groups experienced significant decreases in total hip trabecular vBMD and total hip aBMD, ranging from −1.2% to −1.9%. No treatment difference was observed between the WL+VEST and WL groups for total hip trabecular vBMD (estimated treatment difference, +0.91 mg/cm^3^; 97.5% CI, −0.27 to 2.09 mg/cm^3^; *P* = .13) or total hip aBMD (estimated treatment difference, +1.93 mg/cm^2^; 97.5% CI, −7.66 to 11.52 mg/cm^3^; *P* = .67). WL+VEST was noninferior to WL+RT with respect to 12-month change in total hip trabecular vBMD (estimated treatment difference, +0.29 mg/cm^3^; 98.75% lower bound only, −1.05 mg/cm^3^) and total hip aBMD (estimated treatment difference, +0.83 mg/cm^2^; 98.75% lower bound only, −10.08 mg/cm^3^). Group by time data for primary and prespecified secondary outcomes are displayed graphically in Figure 2.

**Table 2.** Primary and Prespecified Secondary Outcomes by Treatment Group^a^

Outcome measure	Month	WL		WL+VEST		WL+RT		Treatment effects		
								WL+VEST vs WL change, mean (97.5% CI)		WL+VEST vs WL+RT change, mean (98.75% lower bound only)
		Change, mean (95% CI)	Change, %	Change, mean (95% CI)	Change, %	Change, mean (95% CI)	Change, %			
Total hip trabecular vBMD, mg/cm^3^	6	−0.50 (−1.24 to 0.24)	−0.4	−0.66 (−1.40 to 0.07)	−0.5	−0.13 (−0.87 to 0.61)	−0.1	−0.17 (−1.04 to 0.71)		−0.53 (−1.40)
	12	−2.52 (−3.50 to −1.54)	−1.9	−1.61 (−2.58 to −0.64)	−1.2	−1.90 (−2.87 to −0.93)	−1.4	0.91 (−0.27 to 2.09)		0.29 (−1.05)^a^
Total hip aBMD, mg/cm^2^	6	−8.03 (−15.17 to −0.89)	−0.8	−5.71 (−12.79 to 1.38)	−0.6	−7.95 (−15.20 to −0.70)	−0.8	2.32 (−6.11 to 10.75)		2.24 (−6.13)
	12	−16.93 (−25.00 to −8.87)	−1.7	−15.00 (−22.98 to −7.03)	−1.5	−15.84 (−23.97 to −7.71)	−1.5	1.93 (−7.66 to 11.52)		0.83 (−10.08)^b^

Abbreviations: aBMD, areal bone mineral density; vBMD, volumetric bone mineral density; WL, weight loss; WL+RT, weight loss plus resistance training; WL+VEST, weight loss plus weighted vest.

^a^ All follow-up estimates were derived from a mixed-effects linear model fit using the treatment effect, visit, treatment × visit interaction, gender, hip side (qualitative computed tomography only, right/left), and baseline values of each outcome as fixed effects and participant identification numbers as random effects. Contrast statements were used to derive group mean estimates, treatment effects, CIs, and *P* values at 6 and 12 months. Pairwise comparisons and confidence bounds at 12 months assumed a type 1 error rate (2-sided CI) of 0.025 for WL+VEST vs WL only and type 1 error rate of 0.0125 (lower bound only) for WL+VEST vs WL+RT. All visit-specific means and change estimates are presented as 95% CIs.

^b^ The prespecified noninferiority margin between WL+VEST and WL+RT for 12-month difference in total hip trabecular vBMD groups was based on a 98.75% lower bound for the difference that does not exceed −4% of RT (0.130 g/cm^3^ [−0.04] = −0.0052). The observed bound (−0.001 g/cm^3^) does not exceed the value, establishing that WL+VEST is noninferior to WL+RT with respect to total hip trabecular vBMD.

^c^ The prespecified noninferiority margin between WL+VEST and WL+RT for 12-month difference in total hip aBMD groups was based on a 98.75% lower bound for the difference that does not exceed −2.13% of RT (1.009 g/cm^2^ [−0.0213] = −0.0215). The observed bound (−0.010 cm^2^) does not exceed the value, establishing that WL+VEST is noninferior to WL+RT with respect to total hip aBMD.

Additional secondary and ad hoc outcome measures are presented by treatment group in Table 3. No significant treatment effects were observed for any CT or DXA skeletal outcome except for a slight elevation in mean L1 to L4 trabecular vBMD at 12 months in the WL+RT group (change, +2.74 mg/cm^3^; 95% CI, 0.60 to 4.87 mg/cm^3^) compared with the WL (change, +0.10 mg/cm^3^; 95% CI, −2.03 to 2.22 mg/cm^3^) and WL+VEST (change, −0.08 mg/cm^3^; 95% CI, −2.17 to 2.01 mg/cm^3^) groups (*P* = .049). A significant treatment effect was observed for DXA-acquired total body fat mass, where greater 12-month losses were reported for the WL+RT group (change, −9.99 kg; 95% CI, −11.65 to −8.33 kg) compared with the WL (change, −7.69 kg; 95% CI, −9.35 to −6.03 kg) and WL+VEST (change, −7.31 kg; 95% CI, −8.95 to −5.66 kg) groups (*P* = .02). Statistically significant total body lean mass loss was observed in all groups, representing a 1.5% to 1.9% change from baseline and approximately 8% of total weight lost. No difference in a marker of bone resorption (C-terminal telopeptide) was noted between groups; however, the bone formation biomarker (P1NP) was increased by 12 months in the WL+VEST (change, +3.12 μg/L; 95% CI, −1.22 to 7.46 μg/L) and WL+RT (change, +2.29 μg/L; 95% CI, −2.03 to 6.61 μg/L) groups compared with WL alone (change, −3.31 μg/L; 95% CI, −8.02 to 1.39) (*P* = .02). Finally and as expected, 12-month knee extensor strength and self-reported moderate to vigorous physical activity were elevated in the WL+RT group (change, +5.52 N · m; 95% CI, 1.02 to 10.02 N · m; and change, +2571.00 kcal/wk; 95% CI, 1927.17 to 3214.83 kcal/wk, respectively) compared with the WL+VEST (change, −0.47 N · m; 95% CI, −5.25 to 4.30 N · m; and change, +1445.61 kcal/wk; 95% CI, 806.21 to 2085.01 kcal/wk, respectively) and WL (change, −2.26 N · m; 95% CI, −6.90 to 2.37 N · m; and change, +1553.86 kcal/wk; 95% CI, 904.07 to 2203.65 kcal/wk, respectively) groups (*P* = .01).

**Table 3.** Secondary and Ad Hoc Outcomes by Treatment Group^a^

Outcome measure	Month	WL		WL+VEST		WL+RT		*P* value
		Change, mean (95% CI)	Change, %	Change, mean (95% CI)	Change, %	Change, mean (95% CI)	Change, %	
CT-based measures								
Total hip cortical vBMD, mg/cm^3^	6	5.92 (2.25 to 9.60)	0.8	9.41 (5.76 to 13.05)	1.3	5.47 (1.79 to 9.14)	0.8	.15
	12	8.20 (4.00 to 12.41)	1.2	6.89 (2.69 to 11.09)	1.0	6.56 (2.36 to 10.75)	0.9	.79
Femoral neck trabecular vBMD, mg/cm^3^	6	−0.63 (−2.43 to 1.16)	−0.5	−1.16 (−2.93 to 0.60)	−0.9	−0.86 (−2.65 to 0.93)	−0.7	.89
	12	−4.25 (−6.50 to −2.00)	−3.3	−1.71 (−3.96 to 0.53)	−1.3	−2.73 (−4.97 to −0.50)	−2.1	.18
Femoral neck cortical vBMD, mg/cm^3^	6	9.79 (4.20 to 15.38)	1.4	6.68 (1.18 to 12.19)	1.0	7.19 (1.60 to 12.79)	1.0	.61
	12	11.63 (6.06 to 17.21)	1.7	9.28 (3.77 to 14.80)	1.3	13.09 (7.50 to 18.69)	1.9	.52
L1-L4 mean trabecular vBMD, mg/cm^3^	6	4.81 (1.57 to 8.05)	4.0	1.42 (−1.76 to 4.59)	1.2	4.58 (1.35 to 7.80)	3.8	.16
	12	0.10 (−2.03 to 2.22)	0.1	−0.08 (−2.17 to 2.01)	−0.1	2.74 (0.60 to 4.87)	2.3	.05
DXA-based measures								
Femoral neck aBMD, mg/cm^2^	6	4.23 (−6.63 to 15.10)	0.4	−0.44 (−11.14 to 10.25)	−0.0	3.53 (−7.42 to 14.48)	0.4	.74
	12	−4.78 (−16.08 to 6.53)	−0.5	−6.01 (−17.12 to 5.11)	−0.6	−3.84 (−15.20 to 7.52)	−0.4	.95
Distal radius aBMD, mg/cm^2^	6	−6.84 (−15.89 to 2.22)	−0.8	−9.19 (−18.49 to 0.11)	−1.0	−8.48 (−17.63 to 0.68)	−1.0	.91
	12	−12.60 (−22.39 to −2.81)	−1.4	−12.52 (−22.49 to −2.55)	−1.4	−7.20 (−17.05 to 2.66)	−0.8	.57
Lumbar spine aBMD, mg/cm^2^	6	15.53 (1.15 to 29.90)	1.2	3.15 (−11.18 to 17.47)	0.2	14.94 (0.16 to 29.71)	1.2	.27
	12	9.66 (−8.04 to 27.36)	0.8	5.84 (−11.71 to 23.39)	0.5	10.52 (−7.41 to 28.46)	0.8	.90
Trabecular bone score	6	0.017 (−0.002 to 0.035)	1.2	0.020 (0.002 to 0.039)	1.4	0.037 (0.018 to 0.056)	2.6	.17
	12	0.017 (−0.003 to 0.037)	1.2	0.014 (−0.005 to 0.034)	1.0	0.037 (0.017 to 0.057)	2.6	.12
Total body fat mass, kg	6	−7.84 (−9.20 to −6.48)	−19.1	−7.80 (−9.14 to −6.45)	−19.0	−8.84 (−10.20 to −7.47)	−21.6	.35
	12	−7.69 (−9.35 to −6.03)	−18.8	−7.31 (−8.95 to −5.66)	−17.8	−9.99 (−11.65 to −8.33)	−24.4	.02
Total body lean mass, kg	6	−0.69 (−1.23 to −0.14)	−1.4	−0.53 (−1.09 to 0.02)	−1.1	−0.81 (−1.38 to −0.23)	−1.7	.67
	12	−0.93 (−1.46 to −0.40)	−1.9	−0.71 (−1.25 to −0.17)	−1.5	−0.86 (−1.42 to −0.30)	−1.8	.76
Blood-based biomarkers^b^								
CTX, μg/L	6	0.05 (0.01 to 0.09)	23.3	0.02 (−0.02 to 0.06)	10.3	0.03 (−0.01 to 0.07)	14.4	.62
	12	0.04 (0.00 to 0.08)	19.2	0.03 (−0.01 to 0.07)	13.9	0.05 (0.01 to 0.09)	22.5	.88
P1NP, μg/L	6	−0.97 (−5.03 to 3.09)	−2.7	0.10 (−3.80 to 4.00)	0.3	2.10 (−1.76 to 5.96)	5.9	.37
	12	−3.31 (−8.02 to 1.39)	−9.4	3.12 (−1.22 to 7.46)	8.8	2.29 (−2.03 to 6.61)	6.5	.02
Ad hoc measures								
Appendicular lean mass, kg	6	−0.73 (−1.12 to −0.34)	−3.2	−0.64 (−1.03 to −0.24)	−2.8	−0.86 (−1.27 to −0.44)	−3.7	.60
	12	−0.93 (−1.32 to −0.54)	−4.1	−0.62 (−1.01 to −0.23)	−2.7	−0.97 (−1.38 to −0.56)	−4.2	.23
Knee extensor strength, N · m	6	0.89 (−3.51 to 5.29)	0.7	−0.97 (−5.58 to 3.63)	−0.8	2.80 (−1.51 to 7.12)	2.4	.36
	12	−2.26 (−6.90 to 2.37)	−1.9	−0.47 (−5.25 to 4.30)	−0.4	5.52 (1.02 to 10.02)	4.6	.01
Moderate to vigorous physical activity, kcal/wk	6	1716.63 (1071.14 to 2362.12)	251.3	1122.60 (486.09 to 1759.11)	164.4	2068.17 (1427.35 to 2709.00)	302.8	.05
	12	1553.86 (904.07 to 2203.65)	227.5	1445.61 (806.21 to 2085.01)	211.7	2571.00 (1927.17 to 3214.83)	376.4	.007

Abbreviations: aBMD, areal bone mineral density; CT, computed tomography; CTX, C-terminal telopeptide; DXA, dual-energy X-ray absorptiometry; P1NP, procollagen 1 intact *N*-terminal propeptide; vBMD, volumetric bone mineral density; WL, weight loss; WL+RT, weight loss plus resistance training; WL+VEST, weight loss plus weighted vest.

^a^ All follow-up estimates were derived from a mixed-effects linear model fit using the treatment effect, visit, treatment × visit interaction, gender, hip side (computed tomography only, right/left), and baseline values of each outcome as fixed effects and participant identification numbers as random effects. *P* values were generated for visit-specific differences among the 3 treatment groups. All visit-specific means and change estimates are presented as 95% CIs.

^b^ Estimates for blood-based biomarkers are presented as least-square means in original scale for interpretability, and *P* values are derived from log-adjusted models.

## Discussion

The purpose of the INVEST in Bone Health trial was to compare the effects of WL alone with WL+VEST or WL+RT on indicators of bone health and subsequent fracture risk among older adults living with obesity. The successful intervention delivery resulted in approximately 10% WL across 3 treatment groups. Participants were largely adherent with weighted vest and RT prescriptions, with the most weighted vest participants reporting that they were satisfied with the weighted vest intervention. Bone loss accompanied dietary WL; however, we did not observe a significant treatment effect of weighted vest use on the primary outcome of 12-month change in hip BMD (assessed via CT or DXA) compared with WL alone. The effect of weighted vest use on hip BMD was noninferior to RT, with both treatments unable to mitigate WL-associated BMD loss. Interpretation of these findings, considering key concepts informing the design and premise of the trial, is presented below.

Clinical studies conducted during the past 30 years provide support for the feasibility and efficacy of weighted vest use to improve skeletal health outcomes. For example, walking while wearing a vest weighted with 5% body mass increased loading on the skeletal system,^37^ and among older women, wearing a weighted vest while exercising (30-60 min/d, 3 d/wk) positively influenced bone turnover^22^ and hip aBMD.^38^ Similarly, wearing a weighted vest around the home improved physical function in older adults and tended to increase BMD.^16^ Notably, one long-term study^18^ showed high adherence (>83%; providing good evidence for participant satisfaction) to weighted vest exercise classes among older women, which prevented aging-related decreases in aBMD (of 3%-4% during a 5-year period). To our knowledge, the only study^25^ examining the ability of weighted vests to minimize bone loss during lifestyle-based WL in older adults was published by our group in 2017. In this pilot 6-month RCT,^25^ which randomized 37 older adults living with obesity to WL alone (n = 17) vs WL+VEST (n = 20, with the goal of ≥10 h/d of weight time with weight added incrementally based on the amount of weight lost), we observed a signal for attenuated loss in total hip aBMD (−0.6% vs −2.0%; *P* = .08) and increased bone formation (bone alkaline phosphatase: +3.8% vs −4.6%; *P* = .07) with weighted vest use.

In comparison to these pilot data, the INVEST in Bone Health trial lengthened the intervention duration to ensure adequate time for bone remodeling^39^ and better align with clinical practice monitoring.^40^ The trial also included sophisticated CT-derived measures of bone, including the primary outcome of total hip trabecular vBMD, which was selected due to its ability to predict hip fractures as well as total hip aBMD in older adults, with superior sensitivity to treatment-related changes and robustness to obesity and WL-induced measurement error.^41^ Indeed, an 18-month RCT from our group, which examined the effect of RT during lifestyle-based WL in older adults living with obesity, noted a more pronounced treatment effect differential on CT-acquired total hip vBMD (−0.026 g/cm^3^ in the WL group vs −0.015 g/cm^3^ in the WL+RT group) compared with DXA-acquired total hip aBMD (−0.023 g/cm^2^ in the WL group vs −0.025 g/cm^2^ in the WL+RT group).^42^ In the current study, a similar degree of bone loss was observed across imaging modalities, with WL-associated changes in CT-acquired vBMD more closely aligning with what has been previously reported for DXA-acquired aBMD (ie, 1%-2% bone loss accompanying 10% WL).^43^

The effect of weighted vest use on hip BMD was noninferior to RT; however, the same degree of hip bone loss was observed in all groups, including the WL-only group. These data contribute to a modest and mixed body of literature evaluating the ability of exercise to diminish loss of bone during WL.^12,13,14^ Treatment effect heterogeneity may be attributed to varying durations and/or training intensities of exercise prescriptions, as well as an underappreciated catabolic effect of exercise on bone,^44^ with RCT data demonstrating the strongest bone-sparing signal with supervised RT.^42,45,46^ In alignment with this observation and our pilot data,^25^ it is noteworthy that P1NP, a marker of bone formation, was increased at 12 months with weighted vest use and RT compared with WL alone. RT also resulted in greater strength gains, which is an important predictor of fall risk.^47^ Because most fractures occur secondary to a fall,^48^ the INVEST in Bone Health intervention effect on muscle function and strength outcomes is an important consideration when interpreting overall skeletal benefit and will be the focus of reports on secondary outcomes.

A final point of consideration is the lower-than-expected bone loss experienced by the WL-only group. The study was powered to observe a 2.5% treatment differential in CT-acquired total hip vBMD,^42^ yet loss observed in the WL-only group was only approximately 2%. We speculate that this finding may be due to the dietary prescription, which included provision of nutritionally complete meal replacement products for the duration of the study^49^ because trials manipulating calcium, vitamin D, and protein intake have found that all of these minerals minimize bone loss associated with weight reduction.^50,51,52^ Relatedly, although all groups experienced similar and statistically significant total body lean mass loss, the magnitude of lean mass loss was less (ie, <2% loss; representing approximately 8% of total weight lost) than what is typically reported in lifestyle-based WL interventions, where 10% total body WL could be expected to yield approximately 20% lean body mass loss.^53^ Given the close coupling between muscle and bone,^54^ the lean mass sparing experienced by all INVEST in Bone Health participants may have contributed to null treatment effects on bone outcomes.

### Strengths and Limitations

Strengths of the INVEST in Bone Health trial include its randomized design, novel intervention, diverse sample, and excellent (albeit in some cases, self-reported) protocol adherence. The trial also included a battery of advanced measures of musculoskeletal health not typically included in WL trials. The study also had some limitations. The lack of a weight-stable control group or information on habitual dietary intake data prohibited formal examination of the influence of age on, or diet quality as a countermeasure to, WL-associated bone loss and should be the focus of future work. Additionally, although the intervention was designed with scalability in mind, the INVEST in Bone Health trial was limited to those living near our academic center who were able to regularly attend exercise sessions.

## Conclusions

In this 12-month RCT, neither weighted vest use nor progressive RT was able to mitigate WL-associated bone loss in older adults living with obesity. Given the robust body of epidemiologic literature consistently linking WL in late life with loss of BMD^55,56,57,58,59^ and increased fracture risk,^55,60,61,62^ identification of scalable and effective strategies to minimize WL-associated bone loss in older adults remains an important public health priority. This study highlights the need for alternative or adjunctive strategies to prevent bone loss in older adults experiencing weight loss because exercise may be insufficient on its own.

## Notes

Supplement 1. **eFigure**. Timeline and Primary Outcome Measures Schematic, created with Biorender **eTable 1.** Baseline Demographic and Clinical Characteristics, stratified by Completion Status **eTable 2**. Summary of Adverse Events Occurring after the Start of Intervention by Treatment Group **eTable 3**. Severity, Relation to Intervention, and Expectedness of Adverse Events **eTable 4**. Summary of Serious Adverse Events Occurring after the Start of Intervention by Treatment Group

Supplement 2.
Trial Protocol

Supplement 3.
Data Sharing Statement
