# Errors in Figure Captions, Table 1, and Table 2

**DOI:** 10.1001/jamanetworkopen.2025.29919

**Published:** 2025-08-06

In the Original Investigation titled “Weighted Vest Use or Resistance Exercise to Offset Weight Loss–Associated Bone Loss in Older Adults: A Randomized Clinical Trial,”^1^ published June 20, 2025, there were errors in the definition of the acronym *WL+RT* in the captions of Figures 1 and 2 and the footnotes of Tables 1 and 2. WL+RT should have been defined as weight loss plus resistance training. There was also an error in the units listed for DXA-acquired areal bone mineral density in Table 1, which should have been mg/cm^2^. This article has been corrected.^1^
