# Supplementary material for: Weighted Vest Use or Resistance Exercise to Offset Weight Loss–Associated Bone Loss in Older Adults: A Randomized Clinical Trial
Source: JAMA Netw Open. 2025 Jun 20;8(6):e2516772. doi: 10.1001/jamanetworkopen.2025.16772 (PMC12181796; doi:10.1001/jamanetworkopen.2025.16772)
Supplement: Supplement 1. — eFigure. Timeline and Primary Outcome Measures Schematic, created with Biorender eTable 1. Baseline Demographic and Clinical Characteristics, stratified by Completion Status eTable 2. Summary of Adverse Events Occurring after the Start of Intervention by Treatment Group eTable 3. Severity, Relation to Intervention, and Expectedness of Adverse Events eTable 4. Summary of Serious Adverse Events Occurring after the Start of Intervention by Treatment Group [file jamanetwopen-e2516772-s001.pdf]

# Supplemental Online Content

Beavers KM, Lynch SD, Fanning J, et al. Weighted vest use or resistance exercise to offset weight loss–associated bone loss in older adults: a randomized clinical trial. *JAMA Netw Open*. 2025;8(6):e2516772. doi:10.1001/jamanetworkopen.2025.16772

**eFigure**. Timeline and Primary Outcome Measures Schematic, created with Biorender

**eTable 1.** Baseline Demographic and Clinical Characteristics, stratified by Completion Status

**eTable 2**. Summary of Adverse Events Occurring after the Start of Intervention by Treatment Group

**eTable 3**. Severity, Relation to Intervention, and Expectedness of Adverse Events

**eTable 4**. Summary of Serious Adverse Events Occurring after the Start of Intervention by Treatment Group

This supplemental material has been provided by the authors to give readers additional information about their work.

© 2025 Beavers KM et al. *JAMA Netw Open*.

**eFigure 1**. Timeline and Primary Outcome Measures Schematic, created with Biorender

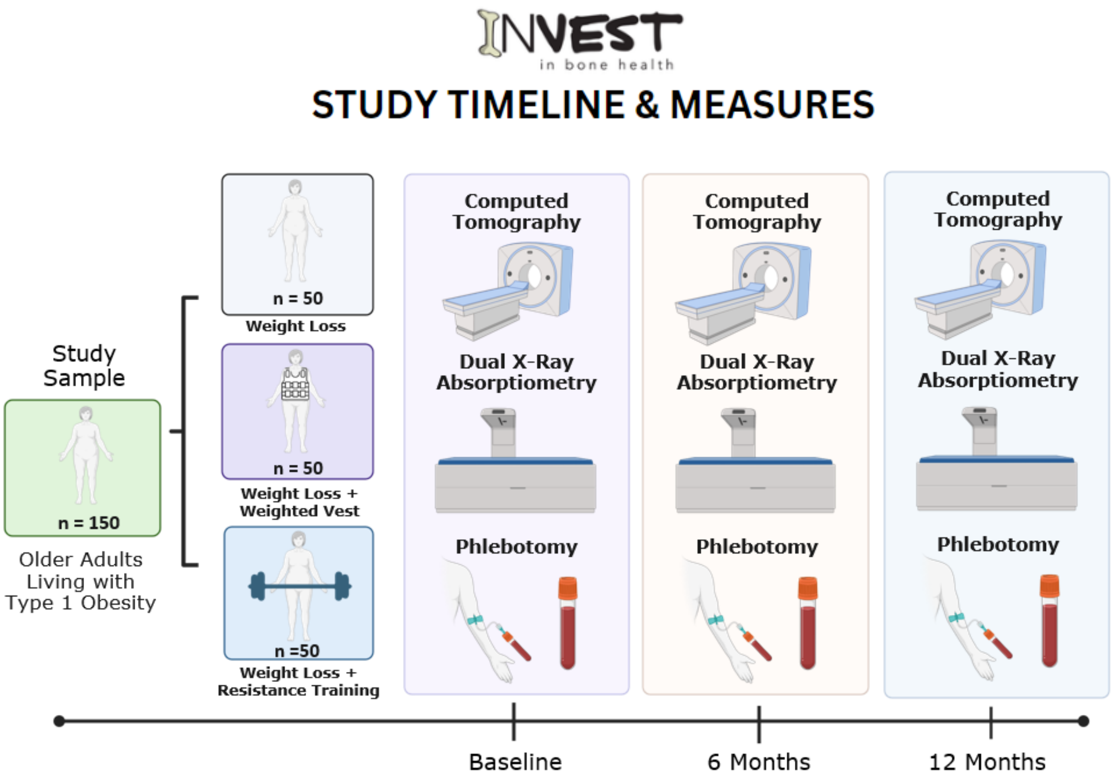


© 2025 Beavers KM et al. *JAMA Netw Open*.

**eTable 1.** Baseline Demographic and Clinical Characteristics, stratified by Completion Status

| | Completers (N = 138) | Non-Completers (N = 12) | p-value |
|---|---|---|---|
| **Age, y.** | 66.36 (4.71) | 66.5 (3.48) | 0.92 |
| **Sex, No. (%)** | | | 0.30 |
| Male | 37 (26.81) | 1 (8.33) | |
| Female | 101 (73.19) | 11 (91.67) | |
| **Race and Ethnicity, No. (%)** | | | 0.11 |
| African American | 37 (26.81) | 6 (50) | |
| Hispanic or Latino | 3 (2.17) | 0 (0) | |
| White | 95 (68.84) | 5 (41.67) | |
| Other[a] | 3 (2.17) | 1 (8.33) | |
| **Body Mass, kg** | 93.13 (12.89) | 90.09 (11.77) | 0.43 |
| **Height, cm** | 166.37 (8.48) | 163.76 (7.06) | 0.30 |
| **Body Mass Index, kg/m$^2$** | 33.58 (3.29) | 33.61 (3.9) | 0.98 |
| **Education, No. (%)** | | | 0.23 |
| High School/Equivalent (09-12) | 28 (20.29) | 0 (0) | |
| College (13-16) | 73 (52.9) | 8 (66.67) | |
| Post-Graduate | 37 (26.81) | 4 (33.33) | |
| **Comorbidities** | | | |
| Diabetes | 10 (7.25) | 2 (16.67) | 0.25 |
| CVD | 16 (11.59) | 0 (0) | 0.36 |
| Arthritis/Joint Pain | 97 (70.29) | 7 (58.33) | 0.51 |
| **Pre-existing low bone mass[b], No. (%)** | 68 (49.28) | 5 (41.67) | 0.77 |

[a]Participants self-reported race and ethnicity. Four participants reported that they considered their race to be "Multiracial or Other," and no further information was collected.
[b]Pre-existing low bone mass: t-score on hip, spine, forearm radius, or femoral neck DXA can of -1 to -2.49

© 2025 Beavers KM et al. *JAMA Netw Open.*

**eTable 2**. Summary of Adverse Events Occurring after the Start of Intervention by Treatment Group

| | | Overall N = 150 | | By Intervention Arm | | | | | |
|---|---|---|---|---|---|---|---|---|---|
| | | | | WL N = 50 | | WL+VEST N = 50 | | WL+RT N = 50 | |
| **Body System** | **Preferred Term** | **Events (N)** | **Participants (%)** | **Events (N)** | **Participants (%)** | **Events (N)** | **Participants (%)** | **Events (N)** | **Participants (%)** |
| Musculoskeletal | -All Musculoskeletal | 42 | 28 | 6 | 12 | 17 | 34 | 19 | 38 |
| | Back Pain | 10 | 6.7 | 1 | 2 | 6 | 12 | 3 | 6 |
| | Shoulder Pain | 6 | 4 | 1 | 2 | 3 | 6 | 2 | 4 |
| | Pain In Knee | 4 | 2.7 | 0 | 0 | 1 | 2 | 3 | 6 |
| | Osteoporosis | 2 | 1.3 | 1 | 2 | 1 | 2 | 0 | 0 |
| | Pain In Hip | 2 | 1.3 | 0 | 0 | 1 | 2 | 1 | 2 |
| | Rotator Cuff Injury | 2 | 1.3 | 1 | 2 | 1 | 2 | 0 | 0 |
| | Tendonitis | 2 | 1.3 | 1 | 2 | 1 | 2 | 0 | 0 |
| | Achilles Tendonitis | 1 | 0.7 | 0 | 0 | 0 | 0 | 1 | 2 |
| | Ankle Sprain | 1 | 0.7 | 0 | 0 | 0 | 0 | 1 | 2 |
| | Ankle Swelling | 1 | 0.7 | 0 | 0 | 0 | 0 | 1 | 2 |
| | Bone Spur | 1 | 0.7 | 0 | 0 | 1 | 2 | 0 | 0 |
| | Fractured Thumb | 1 | 0.7 | 0 | 0 | 0 | 0 | 1 | 2 |
| | Groin Pain | 1 | 0.7 | 0 | 0 | 0 | 0 | 1 | 2 |
| | Hammer Toe | 1 | 0.7 | 0 | 0 | 0 | 0 | 1 | 2 |
| | Low Back Pain | 1 | 0.7 | 0 | 0 | 0 | 0 | 1 | 2 |
| | Neck Strain | 1 | 0.7 | 0 | 0 | 1 | 2 | 0 | 0 |
| | Other, Toe Fusion | 1 | 0.7 | 1 | 2 | 0 | 0 | 0 | 0 |
| | Pain In Extremity | 1 | 0.7 | 0 | 0 | 0 | 0 | 1 | 2 |
| | Pain In Leg | 1 | 0.7 | 0 | 0 | 1 | 2 | 0 | 0 |
| | Rotator Cuff Tear | 1 | 0.7 | 0 | 0 | 0 | 0 | 1 | 2 |
| | Wrist Pain | 1 | 0.7 | 0 | 0 | 0 | 0 | 1 | 2 |
| Gastrointestinal | -All Gastrointestinal | 12 | 8 | 2 | 4 | 3 | 6 | 7 | 14 |
| | Acute Diverticulitis | 2 | 1.3 | 0 | 0 | 1 | 2 | 1 | 2 |
| | Constipation | 2 | 1.3 | 0 | 0 | 1 | 2 | 1 | 2 |
| | Vomiting | 2 | 1.3 | 0 | 0 | 0 | 0 | 2 | 4 |
| | Abdominal Pain | 1 | 0.7 | 0 | 0 | 0 | 0 | 1 | 2 |
| | Bloating | 1 | 0.7 | 1 | 2 | 0 | 0 | 0 | 0 |
| | Diarrhea | 1 | 0.7 | 0 | 0 | 0 | 0 | 1 | 2 |
| | Gastric Ulcer | 1 | 0.7 | 0 | 0 | 0 | 0 | 1 | 2 |
| | Hypercalcemia | 1 | 0.7 | 1 | 2 | 0 | 0 | 0 | 0 |
| | Inguinal Hernia | 1 | 0.7 | 0 | 0 | 1 | 2 | 0 | 0 |
| Cardiovascular | -All Cardiovascular | 3 | 2 | 0 | 0 | 3 | 6 | 0 | 0 |
| | Hypotension | 1 | 0.7 | 0 | 0 | 1 | 2 | 0 | 0 |
| | Sinus Bradycardia | 1 | 0.7 | 0 | 0 | 1 | 2 | 0 | 0 |
| | Syncope | 1 | 0.7 | 0 | 0 | 1 | 2 | 0 | 0 |
| Other | -All Other | 136 | 86.7 | 45 | 88 | 41 | 78 | 50 | 94 |
| | Fall | 31 | 18.7 | 10 | 20 | 14 | 24 | 7 | 12 |
| | Other, Covid-19 Infection | 21 | 13.3 | 5 | 10 | 5 | 10 | 11 | 20 |
| | Sinusitis | 11 | 6.7 | 7 | 12 | 2 | 4 | 2 | 4 |
| | Urinary Tract Infection | 7 | 4 | 1 | 2 | 2 | 4 | 4 | 6 |
| | Cataract Extraction | 3 | 2 | 0 | 0 | 2 | 4 | 1 | 2 |
| | Conjunctivitis | 3 | 2 | 1 | 2 | 0 | 0 | 2 | 4 |

© 2025 Beavers KM et al. *JAMA Netw Open*.

| | | Overall N = 150 | | By Intervention Arm | | | | | |
|---|---|---|---|---|---|---|---|---|---|
| | | | | WL N = 50 | | WL+VEST N = 50 | | WL+RT N = 50 | |
| **Body System** | **Preferred Term** | **Events (N)** | **Participants (%)** | **Events (N)** | **Participants (%)** | **Events (N)** | **Participants (%)** | **Events (N)** | **Participants (%)** |
| | Cough | 3 | 2 | 1 | 2 | 2 | 4 | 0 | 0 |
| | Headache | 3 | 2 | 0 | 0 | 2 | 4 | 1 | 2 |
| | Respiratory Tract Infection | 3 | 2 | 1 | 2 | 0 | 0 | 2 | 4 |
| | Sore Throat | 3 | 2 | 1 | 2 | 1 | 2 | 1 | 2 |
| | Bronchitis | 2 | 1.3 | 0 | 0 | 0 | 0 | 2 | 4 |
| | Cold Symptoms | 2 | 1.3 | 0 | 0 | 1 | 2 | 1 | 2 |
| | Dog Bite | 2 | 1.3 | 1 | 2 | 0 | 0 | 1 | 2 |
| | Influenza | 2 | 1.3 | 0 | 0 | 2 | 4 | 0 | 0 |
| | Influenza Like Illness | 2 | 1.3 | 2 | 4 | 0 | 0 | 0 | 0 |
| | Squamous Cell Carcinoma of Skin | 2 | 1.3 | 1 | 2 | 1 | 2 | 0 | 0 |
| | Abscess | 1 | 0.7 | 1 | 2 | 0 | 0 | 0 | 0 |
| | Allergic Reaction | 1 | 0.7 | 0 | 0 | 1 | 2 | 0 | 0 |
| | Anemia | 1 | 0.7 | 1 | 2 | 0 | 0 | 0 | 0 |
| | Bacterial Eye Infection | 1 | 0.7 | 0 | 0 | 0 | 0 | 1 | 2 |
| | Bell's Palsy | 1 | 0.7 | 1 | 2 | 0 | 0 | 0 | 0 |
| | Breast Mass | 1 | 0.7 | 0 | 0 | 1 | 2 | 0 | 0 |
| | Burn of Unspecified Degree of Back of Hand | 1 | 0.7 | 0 | 0 | 0 | 0 | 1 | 2 |
| | Chest Pain | 1 | 0.7 | 0 | 0 | 0 | 0 | 1 | 2 |
| | Common Cold | 1 | 0.7 | 1 | 2 | 0 | 0 | 0 | 0 |
| | Conjunctivitis Allergic | 1 | 0.7 | 1 | 2 | 0 | 0 | 0 | 0 |
| | Ear Congestion | 1 | 0.7 | 0 | 0 | 1 | 2 | 0 | 0 |
| | Ear Pain | 1 | 0.7 | 1 | 2 | 0 | 0 | 0 | 0 |
| | Epistaxis | 1 | 0.7 | 0 | 0 | 0 | 0 | 1 | 2 |
| | Fever | 1 | 0.7 | 0 | 0 | 0 | 0 | 1 | 2 |
| | Gastroenteritis Viral | 1 | 0.7 | 0 | 0 | 0 | 0 | 1 | 2 |
| | H-Pylori | 1 | 0.7 | 0 | 0 | 1 | 2 | 0 | 0 |
| | Intramuscular Lipoma | 1 | 0.7 | 1 | 2 | 0 | 0 | 0 | 0 |
| | Lichen Planus | 1 | 0.7 | 0 | 0 | 1 | 2 | 0 | 0 |
| | Motor Vehicle Accident | 1 | 0.7 | 0 | 0 | 1 | 2 | 0 | 0 |
| | Nasal Congestion | 1 | 0.7 | 0 | 0 | 0 | 0 | 1 | 2 |
| | Other - Sting | 1 | 0.7 | 1 | 2 | 0 | 0 | 0 | 0 |
| | Other, Cyst | 1 | 0.7 | 0 | 0 | 0 | 0 | 1 | 2 |
| | Other, Low eGFR | 1 | 0.7 | 0 | 0 | 0 | 0 | 1 | 2 |
| | Pancreatic Mass | 1 | 0.7 | 1 | 2 | 0 | 0 | 0 | 0 |
| | Panuveitis | 1 | 0.7 | 0 | 0 | 0 | 0 | 1 | 2 |
| | Pelvic Discomfort | 1 | 0.7 | 0 | 0 | 0 | 0 | 1 | 2 |
| | Platelet Count Decreased | 1 | 0.7 | 1 | 2 | 0 | 0 | 0 | 0 |
| | Poison Ivy Rash | 1 | 0.7 | 0 | 0 | 0 | 0 | 1 | 2 |
| | Rash | 1 | 0.7 | 0 | 0 | 1 | 2 | 0 | 0 |
| | Respiratory Syncytial Virus Infection | 1 | 0.7 | 0 | 0 | 0 | 0 | 1 | 2 |
| | Shingles | 1 | 0.7 | 1 | 2 | 0 | 0 | 0 | 0 |
| | Temporary Vision Loss | 1 | 0.7 | 0 | 0 | 0 | 0 | 1 | 2 |
| | Tooth Infection | 1 | 0.7 | 1 | 2 | 0 | 0 | 0 | 0 |
| | Upper Respiratory Infection | 1 | 0.7 | 1 | 2 | 0 | 0 | 0 | 0 |
| | Vaginal Bleeding | 1 | 0.7 | 1 | 2 | 0 | 0 | 0 | 0 |
| | Vaginal Prolapse | 1 | 0.7 | 0 | 0 | 0 | 0 | 1 | 2 |

© 2025 Beavers KM et al. *JAMA Netw Open*.

**eTable 3**. Severity, Relation to Intervention, and Expectedness of Adverse Events

| | | | Severity | | | | | Relation to Intervention | | | Expectedness | |
|---|---|---|---|---|---|---|---|---|---|---|---|---|
| Body System | Preferred Term | Total Events | Mild N (%) | Moderate N (%) | Severe N (%) | Life-Threatening or Disabling N (%) | Fatal N (%) | Not Related N (%) | Possibly Related N (%) | Definitely Related N (%) | Expected N (%) | Unexpected N (%) |
| Musculoskeletal | Back Pain | 10 | 6 (60%) | 4 (40%) | | | | 5 (50%) | 5 (50%) | | 8 (80%) | 2 (20%) |
| Musculoskeletal | Shoulder Pain | 6 | 5 (83.3%) | 1 (16.7%) | | | | 1 (16.7%) | 5 (83.3%) | | 4 (66.7%) | 2 (33.3%) |
| Musculoskeletal | Pain In Knee | 4 | 3 (75%) | 1 (25%) | | | | 2 (50%) | 2 (50%) | | 3 (75%) | 1 (25%) |
| Musculoskeletal | Osteoporosis | 2 | 2 (100%) | | | | | | 2 (100%) | | 2 (100%) | |
| Musculoskeletal | Pain In Hip | 2 | 1 (50%) | 1 (50%) | | | | | 2 (100%) | | 1 (50%) | 1 (50%) |
| Musculoskeletal | Rotator Cuff Injury | 2 | | | 2 (100%) | | | 2 (100%) | | | | 2 (100%) |
| Musculoskeletal | Tendonitis | 2 | 1 (50%) | 1 (50%) | | | | 2 (100%) | | | | 2 (100%) |
| Musculoskeletal | Achilles Tendonitis | 1 | | 1 (100%) | | | | | 1 (100%) | | 1 (100%) | |
| Musculoskeletal | Ankle Sprain | 1 | 1 (100%) | | | | | 1 (100%) | | | 1 (100%) | |
| Musculoskeletal | Ankle Swelling | 1 | 1 (100%) | | | | | 1 (100%) | | | | 1 (100%) |
| Musculoskeletal | Bone Spur | 1 | | 1 (100%) | | | | 1 (100%) | | | | 1 (100%) |
| Musculoskeletal | Fractured Thumb | 1 | | 1 (100%) | | | | 1 (100%) | | | | 1 (100%) |
| Musculoskeletal | Groin Pain | 1 | 1 (100%) | | | | | | 1 (100%) | | 1 (100%) | |
| Musculoskeletal | Hammer Toe | 1 | | 1 (100%) | | | | 1 (100%) | | | | 1 (100%) |
| Musculoskeletal | Low Back Pain | 1 | 1 (100%) | | | | | 1 (100%) | | | 1 (100%) | |
| Musculoskeletal | Neck Strain | 1 | 1 (100%) | | | | | 1 (100%) | | | | 1 (100%) |
| Musculoskeletal | Other, Toe Fusion | 1 | | 1 (100%) | | | | 1 (100%) | | | | 1 (100%) |
| Musculoskeletal | Pain In Extremity | 1 | 1 (100%) | | | | | | 1 (100%) | | 1 (100%) | |
| Musculoskeletal | Pain In Leg | 1 | 1 (100%) | | | | | | 1 (100%) | | 1 (100%) | |
| Musculoskeletal | Rotator Cuff Tear | 1 | | | 1 (100%) | | | 1 (100%) | | | 1 (100%) | |
| Musculoskeletal | Wrist Pain | 1 | | 1 (100%) | | | | | 1 (100%) | | 1 (100%) | |
| Gastrointestinal | Acute Diverticulitis | 2 | | 2 (100%) | | | | 2 (100%) | | | | 2 (100%) |
| Gastrointestinal | Constipation | 2 | 2 (100%) | | | | | | 1 (50%) | 1 (50%) | 1 (50%) | 1 (50%) |
| Gastrointestinal | Vomiting | 2 | 2 (100%) | | | | | 2 (100%) | | | | 2 (100%) |
| Gastrointestinal | Abdominal Pain | 1 | 1 (100%) | | | | | 1 (100%) | | | | 1 (100%) |

© 2025 Beavers KM et al. *JAMA Netw Open*.

| | | | Severity | | | | | Relation to Intervention | | | Expectedness | |
|---|---|---|---|---|---|---|---|---|---|---|---|---|
| Body System | Preferred Term | Total Events | Mild N (%) | Moderate N (%) | Severe N (%) | Life-Threatening or Disabling N (%) | Fatal N (%) | Not Related N (%) | Possibly Related N (%) | Definitely Related N (%) | Expected N (%) | Unexpected N (%) |
| Gastrointestinal | Bloating | 1 | 1 (100%) | | | | | | 1 (100%) | | 1 (100%) | |
| Gastrointestinal | Diarrhea | 1 | 1 (100%) | | | | | 1 (100%) | | | 1 (100%) | |
| Gastrointestinal | Gastric Ulcer | 1 | | 1 (100%) | | | | 1 (100%) | | | | 1 (100%) |
| Gastrointestinal | Hypercalcemia | 1 | 1 (100%) | | | | | 1 (100%) | | | | 1 (100%) |
| Gastrointestinal | Inguinal Hernia | 1 | | 1 (100%) | | | | 1 (100%) | | | | 1 (100%) |
| Cardiovascular | Hypotension | 1 | | 1 (100%) | | | | | 1 (100%) | | 1 (100%) | |
| Cardiovascular | Sinus Bradycardia | 1 | 1 (100%) | | | | | 1 (100%) | | | | 1 (100%) |
| Cardiovascular | Syncope | 1 | 1 (100%) | | | | | | 1 (100%) | | 1 (100%) | |
| Other | Fall | 28 | 24 (85.7%) | 3 (10.7%) | 1 (3.6%) | | | 23 (82.1%) | 5 (17.9%) | | 3 (10.7%) | 25 (89.3%) |
| Other | Other, Covid-19 Infection | 20 | 20 (100%) | | | | | 20 (100%) | | | | 20 (100%) |
| Other | Sinusitis | 10 | 10 (100%) | | | | | 10 (100%) | | | | 10 (100%) |
| Other | Urinary Tract Infection | 6 | 6 (100%) | | | | | 6 (100%) | | | | 6 (100%) |
| Other | Cataract Extraction | 3 | | 3 (100%) | | | | 3 (100%) | | | | 3 (100%) |
| Other | Conjunctivitis | 3 | 3 (100%) | | | | | 3 (100%) | | | | 3 (100%) |
| Other | Cough | 3 | 3 (100%) | | | | | 3 (100%) | | | | 3 (100%) |
| Other | Headache | 3 | 3 (100%) | | | | | 3 (100%) | | | | 3 (100%) |
| Other | Respiratory Tract Infection | 3 | 3 (100%) | | | | | 3 (100%) | | | | 3 (100%) |
| Other | Sore Throat | 3 | 3 (100%) | | | | | 3 (100%) | | | | 3 (100%) |
| Other | Bronchitis | 2 | 2 (100%) | | | | | 2 (100%) | | | | 2 (100%) |
| Other | Cold Symptoms | 2 | 2 (100%) | | | | | 2 (100%) | | | | 2 (100%) |
| Other | Dog Bite | 2 | 1 (50%) | | 1 (50%) | | | 2 (100%) | | | | 2 (100%) |
| Other | Influenza | 2 | 2 (100%) | | | | | 2 (100%) | | | | 2 (100%) |
| Other | Influenza Like Illness | 2 | 2 (100%) | | | | | 2 (100%) | | | | 2 (100%) |
| Other | Squamous Cell Carcinoma of Skin | 2 | | 2 (100%) | | | | 2 (100%) | | | | 2 (100%) |
| Other | Abscess | 1 | | 1 (100%) | | | | 1 (100%) | | | | 1 (100%) |
| Other | Allergic Reaction | 1 | 1 (100%) | | | | | 1 (100%) | | | | 1 (100%) |

© 2025 Beavers KM et al. *JAMA Netw Open*.

| | | | Severity | | | | | Relation to Intervention | | | Expectedness | |
|---|---|---|---|---|---|---|---|---|---|---|---|---|
| Body System | Preferred Term | Total Events | Mild N (%) | Moderate N (%) | Severe N (%) | Life-Threatening or Disabling N (%) | Fatal N (%) | Not Related N (%) | Possibly Related N (%) | Definitely Related N (%) | Expected N (%) | Unexpected N (%) |
| Other | Anemia | 1 | 1 (100%) | | | | | 1 (100%) | | | | 1 (100%) |
| Other | Bacterial Eye Infection | 1 | 1 (100%) | | | | | 1 (100%) | | | | 1 (100%) |
| Other | Bell's Palsy | 1 | | 1 (100%) | | | | 1 (100%) | | | | 1 (100%) |
| Other | Breast Mass | 1 | | 1 (100%) | | | | 1 (100%) | | | | 1 (100%) |
| Other | Burn Of Unspecified Degree of Back Of Hand | 1 | 1 (100%) | | | | | 1 (100%) | | | | 1 (100%) |
| Other | Chest Pain | 1 | 1 (100%) | | | | | 1 (100%) | | | | 1 (100%) |
| Other | Common Cold | 1 | 1 (100%) | | | | | 1 (100%) | | | | 1 (100%) |
| Other | Conjunctivitis Allergic | 1 | 1 (100%) | | | | | 1 (100%) | | | | 1 (100%) |
| Other | Ear Congestion | 1 | 1 (100%) | | | | | 1 (100%) | | | | 1 (100%) |
| Other | Ear Pain | 1 | 1 (100%) | | | | | 1 (100%) | | | | 1 (100%) |
| Other | Epistaxis | 1 | 1 (100%) | | | | | 1 (100%) | | | | 1 (100%) |
| Other | Fever | 1 | 1 (100%) | | | | | 1 (100%) | | | | 1 (100%) |
| Other | Gastroenteritis Viral | 1 | | 1 (100%) | | | | 1 (100%) | | | | 1 (100%) |
| Other | H-Pylori | 1 | | 1 (100%) | | | | 1 (100%) | | | | 1 (100%) |
| Other | Intramuscular Lipoma | 1 | 1 (100%) | | | | | 1 (100%) | | | | 1 (100%) |
| Other | Lichen Planus | 1 | 1 (100%) | | | | | 1 (100%) | | | | 1 (100%) |
| Other | Motor Vehicle Accident | 1 | 1 (100%) | | | | | 1 (100%) | | | | 1 (100%) |
| Other | Nasal Congestion | 1 | 1 (100%) | | | | | 1 (100%) | | | | 1 (100%) |
| Other | Other - Sting | 1 | 1 (100%) | | | | | 1 (100%) | | | | 1 (100%) |
| Other | Other, Cyst | 1 | | 1 (100%) | | | | 1 (100%) | | | | 1 (100%) |
| Other | Other, Low eGFR | 1 | | 1 (100%) | | | | 1 (100%) | | | | 1 (100%) |
| Other | Pancreatic Mass | 1 | | | 1 (100%) | | | 1 (100%) | | | | 1 (100%) |
| Other | Panuveitis | 1 | 1 (100%) | | | | | 1 (100%) | | | | 1 (100%) |
| Other | Pelvic Discomfort | 1 | | 1 (100%) | | | | 1 (100%) | | | | 1 (100%) |
| Other | Platelet Count Decreased | 1 | 1 (100%) | | | | | 1 (100%) | | | | 1 (100%) |
| Other | Poison Ivy Rash | 1 | 1 (100%) | | | | | 1 (100%) | | | | 1 (100%) |

| | | | Severity | | | | | Relation to Intervention | | | Expectedness | |
|---|---|---|---|---|---|---|---|---|---|---|---|---|
| Body System | Preferred Term | Total Events | Mild N (%) | Moderate N (%) | Severe N (%) | Life-Threatening or Disabling N (%) | Fatal N (%) | Not Related N (%) | Possibly Related N (%) | Definitely Related N (%) | Expected N (%) | Unexpected N (%) |
| Other | Rash | 1 | 1 (100%) | | | | | 1 (100%) | | | | 1 (100%) |
| Other | Respiratory Syncytial Virus Infection | 1 | 1 (100%) | | | | | 1 (100%) | | | | 1 (100%) |
| Other | Shingles | 1 | 1 (100%) | | | | | 1 (100%) | | | | 1 (100%) |
| Other | Temporary Vision Loss | 1 | | 1 (100%) | | | | 1 (100%) | | | | 1 (100%) |
| Other | Tooth Infection | 1 | 1 (100%) | | | | | 1 (100%) | | | | 1 (100%) |
| Other | Upper Respiratory Infection | 1 | 1 (100%) | | | | | 1 (100%) | | | | 1 (100%) |
| Other | Vaginal Bleeding | 1 | | 1 (100%) | | | | 1 (100%) | | | | 1 (100%) |
| Other | Vaginal Prolapse | 1 | | 1 (100%) | | | | 1 (100%) | | | | 1 (100%) |

**eTable 4**. Summary of Serious Adverse Events Occurring after the Start of Intervention by Treatment Group

| Body System | Treatment Arm | Preferred Term | Type of SAE | Association with Intervention | Expected | Severity | Outcome |
|---|---|---|---|---|---|---|---|
| Musculoskeletal | WL+RT | Back Pain | Hospitalization | Not related | Expected | Severe | Recovered with treatment |
| Gastrointestinal | WL | Small Bowel Obstruction | Hospitalization | Not related | Unexpected | Severe | Residual effect(s) present, being treated |
| | WL+VEST | Small Bowel Obstruction | Hospitalization | Not related | Unexpected | Severe | Recovered with treatment |
| Other | WL | Prostate Transurethral Resection | Hospitalization | Not related | Unexpected | Moderate | Recovered with treatment |
| | WL+VEST | Motor Vehicle Accident | Hospitalization | Not related | Unexpected | Severe | Recovered with treatment |
| | | Retinal Detachment | Other serious illness that might have resulted in an SAE without aggressive medical intervention | Not related | Unexpected | Severe | Recovered with treatment |

© 2025 Beavers KM et al. *JAMA Netw Open*.
