# Supplementary material for: Weighted Vest Use or Resistance Exercise to Offset Weight Loss–Associated Bone Loss in Older Adults: A Randomized Clinical Trial
Source: JAMA Netw Open. 2025 Jun 20;8(6):e2516772. doi: 10.1001/jamanetworkopen.2025.16772 (PMC12181796; doi:10.1001/jamanetworkopen.2025.16772)
Supplement: Supplement 2. — Trial Protocol [file jamanetwopen-e2516772-s002.pdf]

Study Title: Incorporating Nutrition, Vests, Education, and Strength Training in Bone Health (INVEST in Bone Health)

Principal Investigator: Kristen M. Beavers, PhD

Co-investigator(s): Barbara J. Nicklas, PhD, Daniel Beavers PhD, Leon Lenchik MD, Ashley A. Weaver PhD, and Jason Fanning, PhD

Sponsor or funding source: National Institutes of Health

Background, Rationale and Context

The prevalence of obesity and its detrimental health effects are increasing rapidly among older adults.[1–5] Medical complications associated with excess fat mass highlight the need to treat obesity in this age group;[6] yet, intentional weight loss (WL) remains controversial.[7–10] Reluctance stems, at least in part, from loss of bone mineral density (BMD) known to accompany overall WL,[11–21] and the potential exacerbation of age-related[22,23] risk of osteoporotic fracture[11,24–28] - a leading cause of injury in older adults that significantly compromises both quality and expectancy of life.[29–32] Importantly, BMD does not appear to be recovered if lost weight is regained,[33–35] and bone loss may even continue at an accelerated rate among those who are successful at long-term WL maintenance.[36] Collectively, these findings provide impetus for better understanding of the mechanisms underlying WL-associated bone loss, particularly among older adults, so that effective intervention strategies can be designed to disrupt them.

Skeletal tissue is highly responsive to mechanical stress;[37] thus, WL-associated declines in mechanical loading likely contribute to BMD loss.[16,19] Clinical trial data consistently demonstrate that the addition of exercise training (designed to enhance gravitational or muscle loading[38–41]) to intentional WL attenuates the amount of bone lost compared to WL alone.[42–46] We recently reported resistance exercise training (RT) is superior to aerobic exercise training in attenuating WL-associated bone *and* muscle losses, while augmenting loss in total body fat mass.[47] However, RT was unable to *fully* prevent bone and muscle loss and, in agreement with prior findings,[48] intervention effectiveness hinged significantly on exercise compliance. Although intuitive, this observation may be especially important for older adults who are less likely[49] (i.e., unwilling or unable) to perform the volume and intensity of exercise necessary to preserve bone during WL. In addition, conventional RT often requires expensive equipment, on-site participation, and (ideally for older adults) safety supervision by trained staff, limiting its scalability as an intervention strategy.

Alternately, treating WL-associated decrease in mechanical load by replacing lost weight externally may also preserve bone mass. In animal models, skeletal metabolism responds in a similar fashion to increases in actual body, or externally added, mass.[50–52] In human studies, wearing weighted vests during exercise improves areal BMD (aBMD) and bone turnover, as well as muscle mass and lower extremity strength.[53–61] Our pilot data suggest this approach is both feasible and likely effective in reducing WL-associated hip aBMD loss by increasing bone formation.[62] If confirmed, the greater availability, ease of administration, and reduced cost associated with weighted vest use to offset WL-associated bone loss, as compared to RT, holds significant public health potential as a translatable strategy to maximize the cardiometabolic benefits of WL, while minimizing potential harm to the musculoskeletal system.

Objectives

The **main goal** of this study is to compare the effects of WL alone with WL plus weighted vest use or WL plus RT on indicators of bone health and subsequent fracture risk. This is a 12 month trial in 150 older (60-85 years) adults with obesity (BMI=30-40 kg/m^2 or BMI= 27.0-<30 kg/m^2 plus one risk factor) randomized to one of three interventions (n= 50/group): WL alone (**WL**; caloric restriction targeting 10% WL with adequate calcium, vitamin D, and protein); WL plus weighted vest use (**WL+VEST;** targeting 8 hours/day, weight replacement titrated up to 10% WL); or, WL plus structured RT (**WL+RT**; targeting 3 sessions/week). In

accordance with recent international position statements on the clinical use of computed tomography (CT) of the hip in the management of osteoporosis,[63,64] total hip trabecular volumetric BMD (vBMD) is the primary outcome. This outcome will be complemented by exploratory assessment of several fracture-related risk factors, including: (1) femoral neck and lumbar spine vBMD, cortical thickness, finite element modeling of bone strength, and regional fat and muscle volumes, measured by CT; (2) aBMD at the total hip, femoral neck, lumbar spine, and distal radius; trabecular bone score; and total body fat/lean masses, measured by dual energy x-ray absorptiometry (DXA); (3) muscle function and strength; (4) biomarkers of bone turnover; and (5) bone-regulating hormones/cytokines known to influence bone metabolism during WL. Therefore, our Specific Aims are to:

Aim 1: Determine the effects of WL+VEST compared to WL and WL+RT on 12 month change in total hip trabecular vBMD. Despite similar reductions in total body weight, ***Hypothesis 1:*** Participants in the WL+VEST group will show attenuated losses of total hip trabecular vBMD versus WL; and ***Hypothesis 2:*** Loss in total hip trabecular vBMD will be no greater in WL+VEST compared to WL+RT.

Aim 2: Explore the effects of WL+VEST compared to WL and WL+RT on the 12 month change in fracture-related risk factors. Despite similar reductions in total body weight, we **hypothesize** that WL+VEST and WL+RT will demonstrate improvements in fracture-related risk factors compared to WL.

Methods and Measures

We will use a three group randomized design in 150 older (60-85 years), obese (BMI=30-40 kg/m^2 or BMI= 27.0-<30 kg/m^2 plus one risk factor), men and women who will undergo a 12 month WL intervention to test our overall hypothesis that weighted vest use added to WL will better preserve bone health compared to WL alone and similarly to WL plus a structured RT intervention. All groups will receive the same WL intervention (caloric restriction targeting 10% WL and with adequate calcium, vitamin D, and protein intake), in accordance with national obesity treatment guidelines.[65] Thus, treatment groups are: WL alone (**WL**; n=50); WL plus weighted vest use (**WL+VEST;** targeting 8 hours/d, weight replacement titrated up to 10% WL; n=50); or, WL plus structured RT (**WL+RT**; targeting 3 sessions/week; n=50); **Table 1**. All outcome assessments occur at baseline, six, and 12 months.

Table 1. Proposed Intervention Components by Treatment Group.

**Intervention Components**	**Intensive WL Phase (Months 1-6)**	**Reduced Contact WL Phase (Months 7-12)**
Weight Loss (**WL**)	Weekly behavioral-based group sessions; 10% WL goal following national obesity treatment guidelines	Biweekly behavioral-based group sessions; 10% WL goal following national obesity treatment guidelines
WL + Weighted Vest (**WL+VEST**)	WL + 8 hours/day weighted vest use titrated weekly to adjust for achieved WL (up to 10% baseline weight)	WL + 8 hours/day weighted vest use titrated biweekly to adjust for achieved WL (up to 10% baseline weight)
WL + Resistance Training (**WL+RT**)	WL + 3 days/week progressive, structured RT	

Recruitment of study participants: Randomization of 150 participants will ensure at least 85% statistical power (with a conservative drop-out rate of ~15%; n=128 completed) to detect significance between group differences in our primary outcome (12 month total hip trabecular vBMD). We plan to recruit, screen and enroll during Years 1-3.5 of the study. We will target older adults who live in the area via media advertisements and mass mailings.

<u>Participant screening and randomization</u>

All individuals who respond to our recruitment strategies will call a phone number and a recruiter will describe the study and perform a brief screening for general eligibility. All participants must conform to the inclusion/exclusion criteria detailed in **Table 2**. <u>These criteria are in place both to verify our target population and to eliminate participants who may be adversely affected by the interventions.</u>

Table 2. Proposed inclusion/exclusion criteria.

Criteria	Inclusion Criteria	Exclusion Criteria	Assessment
Age	Age 60-85 years		Self-report
Obesity status	BMI=30-40 kg/m^2 or<br>BMI 27.0-<30.0 kg/m² AND at least ONE of the following risk factors:<br>1) elevated waist circumference (>35 inches in women, >40 inches in men)<br>2) diabetes,<br>3) hypertension,<br>4) dyslipidemia,<br>5) or other obesity-related comorbidities: clinically manifest coronary artery disease [e.g., history of MI, angina pectoris, coronary artery surgery, coronary artery procedures (e.g., angioplasty) if not within the past year], other atherosclerotic disease [e.g., peripheral arterial disease, abdominal aortic aneurysm, symptomatic carotid artery disease if not within the past year], sleep apnea, or osteoarthritis of the knee or hip (without prescription medication use for at least 3 months within the past year)	Weight greater than 450 lbs (DXA/CT limit)	Measured on scale<br><br>1) Measured with tape measure<br><br>2-4) Self-report and treatment<br><br>5) Self-report
Functional status		Dependent on cane or walker: >2 falls (injurious on non-injurious) in past year	Self-report
Weight Status	No weight loss of >5% in past 6 months	Any contraindications for participation in voluntary weight loss	Self-report
Lifestyle Behaviors		Smoker (>1 cigarette/d or 4/week within year); Drug abuse or excessive alcohol use (>14 drinks/week)	Self-report
Physical status		Participation in regular resistance training and/or high intensity/high impact aerobic exercise for >60 mins per day on > 5 days/week for the past 6 months	Self-report
Cognitive status		Evidence of cognitive impairment (MoCA score <20)	Questionnaire
Orthopedic status	No self-disclosed contraindications for safe and optimal participation in exercise training/vest use.	Osteoporosis (any of the below):<br>Self-reported and on prescription medication.<br>Self-reported prior spine, hip, wrist, or shoulder fracture after age 40 (except when caused by trauma or fall from height).<br>T-score ≤ -2.5 at total hip, femoral neck, lumbar spine, or	Self-report on Medical History form, or medication use or DXA scan

		distal radius scan at screening visit.<br>FRAX 10-year risk scores ≥3% for hip fracture or ≥20% for major osteoporotic fracture (TBS adjusted FRAX is preferable if available).<br><br>Chronic back/shoulder/knee pain with current or past (within 1 year) prescription medication use for at least 3 months.<br><br>Severe, diagnosed arthritis (osteoarthritis, rheumatoid arthritis, or gout) with current or past (within 1 year) prescription medication use for at least 3 months.<br><br>Past (ever) or planned (next 12 months) back surgery.<br><br>Past (6 months prior) or planned (next 12 months) joint replacement surgery.<br><br>Past (ever) unilateral or bilateral hip replacement surgery, or metal device or fixation in hip, pelvis or femur.	
Co-morbidity/health status	Approved for participation by Study Coordinator	Uncontrolled hypertension (BP>160/90 mmHg)<br><br>Current or recent past (within 1 year): severe symptomatic heart disease, uncontrolled angina, stroke, chronic respiratory disease requiring oxygen, neurological or hematological disease requiring treatment for at least 3 months in past year;<br>cancer requiring treatment in the past year (except non-melanoma skin cancer).<br><br>Low vitamin D (<20 ng/mL), abnormal kidney or liver function (2x upper limit of normal), eGFR<45 mL/min/1.73 m^2, Anemia (Hb< 13 g/dL in men/< 12 g/dL in women); Uncontrolled diabetes (fasting glucose >140 mg/dl)	BP measuremen<br><br>Self-report on Medical History Form<br><br>Metabolic panel/ CBC screening blood test
Medication use		Use of growth hormones, weight loss medications, oral steroids, insulin, , or prescription osteoporosis medications in the past year (see medication list).	Self-report on Medical History Form
Technology Status	Willing to complete online/electronic study forms and participate in virtual group sessions, as needed	No home computer, laptop, or tablet with reliable home internet OR no smartphone (touch-screen enabled phone) with reliable unlimited mobile internet.	Self-report
Research participation	Willing to provide informed consent; agree to all study procedures and assessments;<br><br>Able to provide own transit to assessment/intervention visits	Involved in another behavioral/interventional research study, weight loss program or undergoing physical therapy; Unable to tolerate diet, vest, or CT scan (claustrophobia).<br><br>Judged unsuitable for the trial for any reason by clinic staff	Self-report

Schedule of assessment visits

Those who pass phone screening will be scheduled for a screening visit (SV1) which is conducted in the early morning following a 10 hour fast. Before any data collection, participants provide written informed consent and complete a HIPAA authorization form in accordance with our IRB policy. At this visit, weight and height will be measured to calculate BMI. Individuals who meet the BMI criteria will be queried on demographic information and medical history, undergo medical assessments including a blood pressure and pulse check, comprehensive review of medications, and a fasting blood draw (comprehensive metabolic panel

and complete blood count); the Center for Epidemiologic Studies Depression Scale (CES-D)[66] and the Montreal Cognitive Assessment (MoCA)[66] to ensure participants are not depressed and do not have impaired cognitive function (MoCA score ≥20). Individuals with clinically abnormal results at screening will be referred to their primary care providers with their permission.

Potential participants will also be familiarized with the resistance training protocol and asked to complete a dietary and VEST run-in to determine their ability to comply with the intervention protocols. For the dietary run-in, all participants will be provided with and oriented to the OPTAVIA® Optimal Weight 4 & 2 & 1 Plan® guide, sample meal plans, 3 one-day dietary trackers, a sampling of 12 OPTAVIA "Fuelings" (meal replacement products), 3 one-day Product Satisfaction Forms, asked to follow the OPTAVIA Optimal Weight 4 & 2 & 1 Plan for a three day period occurring over the following week, and report on their willingness to follow the diet plan. For the VEST run-in, participants will be asked to wear an unloaded vest for 3 days, and report on their willingness to wear the vest for 8 hours/day for 12 months, if randomized to that arm (SV2).

Eligible participants will undergo further testing at two additional screening visits (SV2, SV3) prior to randomization (using a web-based system, stratified by sex with random block sizes) and intervention start, with outcome assessments repeated at 6 and 12 month testing sessions (see **Table 3** for a detailed assessment timeline). Briefly, outcome measure assessments include: anthropometric measures, physical function testing (grip strength, leg strength, walking tests, stair climb), resting metabolic rate, accelerometry, imaging (DXA and CT), self-reported fatigue, fatigability, and pain, physical activity, and self-efficacy, and blood storage.

Following state and institutional guidelines, modifications to screening and assessment visits will occur initially, and be conducted with as limited participant contact as possible, to ensure the safety of staff and participants, and to decrease the risk of exposure to COVID-19. Modifications will include dividing SV1 into two separate visits: SV1 and SV1-A. Those who pass SV1, will be scheduled for a remote screening visit (SV1-A) to be conducted by phone. At this remote visit, individuals will be queried on medical history, comprehensive review of medications, physical activity status, and the Center for Epidemiological Studies Depression Scale (CES-D). Additional modifications to assessment visits (SV3, FV2, and FV4) and intervention (INT) will include the administration of certain questionnaires and surveys by phone (see **Table 3** for specific questionnaires and surveys). When permissible by the state and the institution, in-person contact for visits will resume following necessary guidelines. Visits will revert back to the original protocol as described above and in **Table 3**, and these changes will no longer be in effect.

Study Interventions

Contact frequency and goals of each component of the three treatment groups for the intensive WL phase (Months 1-6) and the reduced contact WL phase (Months 7-12) are summarized in **Table 1** above.

Weight Loss Intervention (WL; provided to all groups). All participants will undergo a dietary WL intervention designed to elicit behavioral changes leading to decreased caloric intake sufficient to result in a 10% loss of initial body mass (10 kg for a 100 kg individual). This degree of WL is entirely feasible within this timeframe, and we have prior success in achieving this goal in older adults.[67–69] The WL intervention includes the use of a partial meal replacement (MR) program, group nutrition education, state-of-the-art methods for promoting WL based on the group dynamics literature, social cognitive theory, and strategies that optimize self-regulation.[70–72] All participants will be asked to initially follow the OPTAVIA® Optimal Weight 4 & 2 & 1 Plan®. This is a portion-controlled, reduced calorie (1100-1300 calories/day) weight loss meal plan and includes consumption of a total of 4 OPTAVIA MRs, 2 self-prepared lean and green meals, and 1 Healthy Snack per day. Each of the OPTAVIA MRs contain ~90-110 kcals, 10-15 g protein, are fortified with at least 20% of the daily value for 24 vitamins and minerals (including calcium and vitamin D), and contain GanadenBC30 probiotic cultures. All OPTAVIA MRs share a similar nutritional profile, allowing them to be used interchangeably, and will be shipped directly to participants. Participants will purchase and prepare ingredients for the foods for the lean and green meals on their own. Each lean and green meal will consist of 5-7 oz. lean protein, 3 servings of non-starchy vegetables and up to 2 servings of healthy fat. The healthy snack will consist of a self-selected serving of fruit, dairy, or grain purchased by the participant, or one of OPTAVIA's portable, pre-portioned, ready-to-eat

snacks can be used as a healthy snack. Participants will be guided by the study RD on their food purchasing and preparation of the other meals and will be encouraged to consume only what is approved from the menu.

Once a participant has met or exceeded 10% weight loss for 3 consecutive group session weigh-ins, they will be given the option to transition to the OPTAVIA Lean & Green Life™ maintenance meal plan. Any individual for whom additional weight loss appears to be a safety concern will be directed to transition to the Lean & Green Life Plan immediately. The Lean & Green Life Plan takes into consideration a participant's individual calorie needs (based on total energy expenditure and monitoring of weight), builds on the principles learned during weight loss, and offers flexibility while simultaneously providing boundaries for those who may need them. The Lean & Green Life Plan includes a transitional period during which individuals gradually increase their daily calories and decrease the number of OPTAVIA MRs consumed. At the end of the transitional period, participants will be recommended to eat 3 OPTAVIA MRs, 2 self-prepared lean and green meals (with expanded lean offerings to include more plant-based proteins, such as beans and lentils) and make up the balance of their caloric needs with Healthy 100's (whole food and beverage options derived from the Diabetes Exchange List, each with approximately100 calories and up to 15 g of carbohydrate). A list of such foods/beverages that meet the requirements for Healthy 100's will be provided to participants to facilitate their food choices. Participants who would prefer to decrease or discontinue using the OPTAVIA MRs during maintenance can substitute them with the equivalent number of Healthy 100's (e.g., 1 OPTAVIA MR can be swapped for 1 additional Healthy 100's) while on the Lean & Green Life Plan.

In addition to receiving dietary counseling, all participants will be asked to attend virtual or in-person behavioral counseling group sessions led by the RD. These sessions will be held weekly for the first 6 months and bi-weekly for the remainder of the study to provide participants an opportunity to review specific questions and problems. The education and counseling component of the intervention will emphasize instruction in making healthy, lower-calorie food choices while teaching changes in behavioral habits to promote WL and prevent weight regain.[73] Specific emphasis will be placed on the acquisition and use of self-management skills, particularly self-monitoring of diet and weight, and achieving 150 minutes of aerobic activity/week, in accordance with national guidelines.[65,74] Throughout the 12 month WL phase, participants will be asked to track their food/beverage intake (including the number of meal replacements consumed each day) daily for the first three months and then on a reduced schedule as warranted. These will be reviewed routinely by the study registered dietitian. Body weight will be measured and recorded at all group sessions to provide additional feedback and to increase motivation. Official study weights will occur at SV3, FV2, and FV4. If it is evident (either through review of diet records or inadequate WL) that participants are struggling to meet their WL goals additional individual counseling sessions with the RD will be held to improve compliance. At FV1 and FV3, participants in this group will be asked to complete a program evaluation form to solicit feedback on overall WL program satisfaction, desired future usage, and product preferences. Periodically throughout intervention, participants will be asked to rate their confidence (i.e., self-efficacy) in meeting the demands of the intervention they are receiving. They will also be asked to report changes in their health and medication.

Weight Loss + Weighted Vest Intervention (WL+VEST). The WL component of the intervention is described above. Participants assigned to the WL+VEST group will each receive an appropriately-sized weighted vest for the duration of the 12 month intervention. The vest fabric and design fits comfortably over or under clothing (individual weights are ¼ inch thick), allows for full range of motion and movement, and full chest expansion without restricting breathing. An unloaded vest weighs about one pound and is available in several sizes, including XXL. The vest firmly and evenly distributes weight over the body's core when the small (about the size of a domino piece) 2.25 oz weight blocks are added into the vest pockets. Participants in this group will be asked to wear the vest on a daily basis, progressing to a goal of 8 hours/day (by week 2) during their most active part of the day. Initially, the vest will be unloaded (e.g., one-pound vest only without added blocks). The vest weight will then be incremented according to each participant's rate of WL based on their weekly (first 6 months) and biweekly (final 6 months) body weight assessments. As body mass is lost, the weight of the vest will be increased (with the 2.25 oz weight blocks) to replace the lost weight. The goal is to maintain the gravitational loading on the musculoskeletal system throughout the intervention by replacing lost body mass via

external loading. To objectively monitor wear time, intervention staff will periodically embed a small accelerometer into one of the vest pockets during the weekly/biweekly group sessions. These devices collect accelerations across the X, Y, and Z axes and conventional wear-time algorithms will be applied to identify periods of non-wear time of the vest. Participants will also keep a daily log to record the time worn, vest weight, and any complications or comments related to the vest use, with overall satisfaction of vest use assessed at study close. Intervention staff will monitor and discuss accelerometer output and daily logs at the end of each group session, intervening to enhance compliance to the vest protocol when necessary.

Weight Loss + Structured Resistance Exercise Training Intervention (WL+RT). The WL component of the intervention is described above. Participants assigned to the WL+RT group will undergo a progressive RT intervention designed to elicit adaptations in the musculoskeletal system. Exercise sessions will take place on three non-consecutive days/week, and will be supervised by staff exercise trainer also trained in basic life support and emergency management procedures. The RT program is based on recent guidelines with respect to intensity, number of repetitions, exercises, sets, and days per week.[78] The maximal weight that can be lifted with correct form in a single repetition (1RM) will be used to prescribe intensity. Progression of intensity, repetitions and sets will be individualized and gradual to allow participants to familiarize themselves with the equipment, minimize muscle soreness and reduce potential for injury. The training goal is for participants to complete three sets of 10-12 repetitions for 8 different exercises at 70-75% 1RM for that given exercise. Participants will be instructed to rest for approximately one minute between each set. The resistance will be incremented when a participant can complete 12 repetitions for two of three sets on two consecutive sessions. Heart rate and blood pressure will be measured before and after each session. Participants will be instructed to warm-up at the start of each session by walking or cycling for 5-10 minutes at a slow pace, and will end each session with a cool-down of light stretching. The interventionists will ensure that participants adjust the equipment appropriate to their body size and complete the exercises with correct form.

Methods for Study Assessments

Table 3 provides a detailed study assessment timeline. The nature, purpose, and risks of all procedures and protocols will be explained to each participant prior to obtaining written consent. All examiners are trained in the standardized conduct of all assessments before data collection. Participants will be instructed to wear appropriate and comfortable clothing, and standardized written instructions will be provided prior to each study visit.

INVEST Measurements	PS	SV1	SV1-A	SV2	SV3	INT	FV1	FV2	INT	FV3	FV4	SV3, INT, FV2, FV4
Participant Status		fasting			fasting			fasting			fasting	
Weeks		-8 to 0				1-24	22-26		25-52	50-54		
Location		WFSM or WFU	Phone	WFU	WFSM	WFSM or WFU	WFU	WFSM	WFSM or WFU	WFU	WFSM	Phone
**Questionnaires**												
Phone Screener	x											
Informed consent/HIPAA		x										
Demographics		x										
Medical history		x	x									
Medications		x	x	x	x		x	x		x	x	x
Depression (CES-D)		x	x				x			x		x
Cognitive Assessment (MoCA)		x								x		
Pittsburgh Fatigability Scale					x		x			x		x
PROMIS Pain Intensity and Fatigue					x		x			x		x
CHAMPS physical activity		x	x				x			x		x
Adverse Events				x	x	x	x	x	x	x	x	x
**Physical Exams and Physical Performance Measures**												
Vital signs (ht/wt/bp/pulse)		x		x	x		x	x		x	x	
Anthropometric Measures				x			x			x		
eSPPB				x			x			x		
Hand Grip Test				x			x			x		
RMR					x			x			x	
Activity Monitor - thigh				x			x			x		
Activity Monitor - in vest						x			x			
400 meter Walk – fast				x			x			x		
TUG				x			x			x		
Biodex					x			x			x	
Stair climb				x			x			x		
**Radiology/Imaging Tests**												
DXA (whole body, hip, spine, forearm)				x			x			x		
CT (hip and spine)					x			x			x	
**Phlebotomy**												
CBC		x										
Metabolic Panel		x										
Vitamin D					x							
Plasma/Serum Storage					x			x			x	
**Other**												
D3-Creatine muscle mass (dose/collection)				x	x					x	x	
Run-in instructions		x		x								
Participant Satisfaction									x			x
Self-Efficacy						x			x			x
Adherence monitoring (weight, dietary trackers, meet with study dietitian as needed).						x			x			

Table 3. Proposed Assessment Timeline.

PS=phone screen, SV=screening visit, FV=follow up visit, INT=intervention; eSPPB =expanded short physical performance battery, RMR=resting metabolic rate, TUG=timed up and go, DXA=dual energy x-ray absorptiometry, CT=computed tomography, CBC=complete blood count.

Study Measure(s)

Methods for Primary Outcome Measure Assessment. Our study is powered to detect group differences in our primary outcome of 12 month change in total hip trabecular vBMD measured from CT scans acquired at baseline and 12-month follow-up visits. CT scans will also be acquired at a 6-month visit to assess changes in the outcomes at the end of the intensive WL phase. For completeness, below we describe the CT scanning protocol and vBMD image acquisition and assessment methods for the total hip, femoral neck, and lumbar spine regions of interest.

CT Scanning Protocol. A helical CT scan including the femurs and lumbar spine will be obtained at baseline, 6, and 12 months on a 64-slice scanner (PET/CT GE Discovery MI scanner housed within the WFSM Translational Imaging Program). Scans will be acquired with SFOV 50 cm and 120 kV with standard reconstruction and secondary reconstruction using a bone algorithm. The scan will cover from the top of L1 through 3 cm below the mid-shaft of the femurs (mid-shaft defined as the midpoint between the superior aspect of the greater trochanter and the inferior aspect of the lateral condyle). The 5-port bone mineral calibration phantom (Mindways CT, Austin, TX) will be imaged in every scan and must be positioned to cover the lumbar spine through the lesser trochanter. In rare instances where the calibration phantom does not properly cover these regions, a scan may need to be repeated for proper data collection. The repeat scan will add approximately 975 millirem of radiation or less to the participant's radiation dose. Participants will have the option to decline a repeat scan, if it is determined that one is needed. In an effort to avoid repeated scans, trained study staff are present at each scan to ensure that the CT protocol is administered correctly. However, there may be times where study staff are not able to be present, and a re-scan may be needed if the scan is not acquired properly during these instances. Quality assurance of the CT system will be performed according to manufacturer specifications.

vBMD Acquisition and Assessment. vBMD of the total hip, femoral neck, and lumbar spine will be obtained using QCT Pro^{TM} software (Mindways, Austin, TX). The software automatically segments the proximal femur to obtain total hip trabecular vBMD (primary outcome measure), and total hip cortical and integral vBMD. The proximal femur is also automatically divided into anatomical compartments to measure trabecular, cortical, and integral vBMD of the femoral neck. Bilateral femoral vBMD averages will be reported, when possible. Regions of interest (ROIs) are automatically placed within a mid-vertebral slice at each vertebral level to obtain lumbar spine trabecular vBMD. Segmentations and ROIs will be manually adjusted as appropriate. The CT Hounsfield units (HU) are calibrated using the 5-port bone mineral phantom to derive equivalent aqueous potassium phosphate density measures of vBMD in mg/cm^3. Elasticity-density relationships will be used to derive subject-specific material properties from vBMD measurements for inclusion in finite element (FE) models.[75–77]

Muscle cross-sectional area (CSA), muscle density, and intermuscular fat CSA will be measured for the total abdominal muscles and psoas muscle at a single CT slice at the L3 vertebra level. Visceral and subcutaneous fat CSAs will also be measured in this slice. Mid-thigh muscle CSA, muscle density, subcutaneous fat CSA, and intermuscular fat CSA will be assessed at the mid-shaft position defined as the midpoint between the superior aspect of the greater trochanter and the inferior aspect of the lateral condyle measured on an anterior-posterior scout of the entire femur. Muscle and fat areas will be semi-automatically segmented from CT using Mimics software (v19, Materialise, Plymouth, MI) by thresholding for fat (-190 to -30 HU) and muscle (-29 to 150 HU) and then manually refining segmentations

Methods for Exploratory Outcome Measure Assessment. Several exploratory fracture-related risk factors will be assessed, including: (1) CT-acquired femoral neck and lumbar spine vBMD (described above), cortical thickness, FE modeling of bone strength, and regional fat/lean volumes; (2) DXA-acquired total hip, femoral neck, lumbar spine, and distal radius aBMD, trabecular bone score, and total body fat/lean masses; (3) objectively measured muscle function and strength; (4) biomarkers of bone turnover; (5) hormones and

cytokines known to influence bone metabolism during WL; and (6) D3-Creatine derived muscle mass. As a team, we have extensive experience collecting all secondary outcome measures.[47,62,78,79,67,80–88]

Cortical Thickness Acquisition and Assessment. Variable cortical thickness across the entire surface of the proximal femur and lumbar spine will be obtained using an algorithm validated to accurately measure thicknesses as small as 0.3 mm from clinical CT scans (Stradview, Cambridge University, UK).[87,89,90] A mathematical model constrained by a global cortical density and out-of-plane blur is fit to HU intensities measured from a line normal to the cortical surface that passes through the soft tissue, cortex, and trabecula. Point clouds of the inner and outer cortex surfaces are output, as well as cortical thicknesses (~14,000/femur; ~3,000/vertebrae). As we've done previously, a mapping approach will be applied to assign subject-specific cortical thicknesses to each node of the cortical shell elements in the FE models.[87]

FE Modeling to Generate Strength Estimates. Subject-specific FE models of the proximal femur and lumbar spine will be developed using mesh morphing to accelerate model development.[88,91,92] Thin-plate spline radial basis function interpolation and a relaxation algorithm will be used to morph an existing FE model (i.e. atlas) to a subject-specific geometry. Atlases will be the Global Human Body Models Consortium (GHBMC) M50-O v4.4 femur and the Total Human Model for Safety AM50 v4.02 lumbar spine.[88,91–94] Homologous landmarks from analogous locations on the atlas and subject-specific geometries will be used to derive an interpolation function and coefficients to morph the atlas FE model nodal coordinates. Homologous landmarks are collected using image segmentation and registration to derive atlas and subject-specific point clouds.[95] To evaluate the quality of the registration and morphing algorithms, a deviation analysis will be completed to quantify the point-to-surface distances between the subject-specific landmarks and the morphed FE nodal coordinates and the 3D triangulated surface models derived from the CT scan data. The subject-specific FE models will incorporate vBMD-derived material properties and variable cortical thicknesses (see above sections). Bone strength will be estimated through simulation of the following experimental tests: single-limb stance, sideways fall, and quasi-static uniaxial vertebral compression test.[93,94] The peak fracture force or bone strength will be defined as the peak force recorded between the impactor and femoral head or vertebral body.

Regional Fat and Muscle Volumes. Muscle/fat/bone cross-talk is an emerging area of scientific inquiry,[96,97] and because little is known regarding the interaction of regional fat/muscle loss and bone density changes during intentional WL in older adults with obesity, this study positions us to use the CT-acquired data to generate regional fat and muscle volumes, to complement DXA-acquired appendicular lean mass and visceral fat area (see section below). Abdominal visceral and subcutaneous fat volumes will be measured using approximately 60 slices taken within 15 mm centered at the L4–L5 level. Mid-thigh muscle volume and intermuscular fat will also be assessed, with mid-shaft position defined as the midpoint between the superior aspect of the greater trochanter and the inferior aspect of the lateral condyle measured on an anterior-posterior scout of the entire femur. Muscle and fat volumes will be semi-automatically segmented from CT using Mimics software (v19, Materialise, Plymouth, MI) by thresholding for fat (-190 to -30 HU) and muscle (-29 to 150 HU) and then manually refining segmentations.

<u>DXA-Acquired Outcome Measures.</u> Areal BMD of the whole body, total hip, femoral neck, lumbar spine, and distal radius will be determined by dual-energy x-ray absorptiometry (iDXA, GE Medical Systems, Madison, WI). Coefficients of variation (CV) from repeated measurements at our institution are <2% for hip and spine regions. All scans will be performed and analyzed by an International Society for Clinical Densitometry (ISCD) trained DXA technologist, under the supervision of Dr. Lenchik. Every scan will be examined to evaluate for proper patient positioning and analysis, and reanalysis or rescanning will be performed if necessary. Any artifacts will be noted and, if possible, excluded from the measured region. Daily quality control scans will be obtained with a calibration phantom. If results are more than two SD from baseline the phantom scans will be repeated. Additional DXA-acquired measures include trabecular bone score (TBS; %CV: 3.3), obtained from the lumbar spine scans; total body fat mass (%CV: 1.3); total body lean mass (%CV: 0.9); appendicular lean

mass; and visceral fat area (measured from the whole-body scan in a five cm wide region across the entire abdomen just above the iliac crest at a level approximate with the 4th lumbar vertebrae).

Objectively Measured Muscle Function and Strength. Gait speed will be assessed using the **fast 400 meter walk test**. The 400 meter walk tests was originally developed in Health ABC[98] and used by our group in several clinical trials.[70,71,99] Participants are asked to walk 10 laps of a 40 meter course (20 meters out and 20 meters back) as fast as possible. A script is used to standardize instructions to all participants. Participants can stop and rest if necessary, but are not allowed to sit, and are given a maximum of 15 minutes to complete the test. The reliability and validity of the 400 meter walk test are excellent.[100,101] Physical performance will be assessed using the **expanded Short Physical Performance Battery (SPPB)**. The expanded SPPB consists of five repeated chair stands, standing balance (semi- and full-tandem stands and a single leg stand for 30 seconds), a four meter walk to assess usual gait speed, and a narrow four meter walk test of balance (walking at usual pace within lines of tape spaced 20 cm apart).[102] Scores for the traditional 0-12 point SPPB can also be obtained from these tests. We will also assess physical performance using the **Timed-Up-and-Go (TUG)**. TUG measures the time a person takes to stand up from a standard chair, walk three meters, turn, walk back to the chair, and sit down again.[103] A practice trial is given, followed by two timed trials and the results of the timed trials are averaged. **Stair climbing time** will be assessed by using the participant's fastest time achieved to climb 12 steps 12 step staircase in two trials. **Lower extremity muscle strength** will be measured using an isokinetic dynamometer with the participant sitting and the hips and knee flexed at 90°. The dynamometer will be adjusted for each participant and all adjustments will be recorded to duplicate the position for subsequent assessments. Start and stop angles will be set at 90° and 30°. Participants will be asked to extend the knee and push as hard as possible against the resistance pad. Strength is expressed as peak torque (Nm). **Grip strength** will be measured twice in each hand to the nearest two kg using an isometric Jamar Hydraulic Hand Dynamometer (Performance Health, Warrenville, IL) with the mean value from the stronger hand used.

Biomarkers of Bone Turnover and Key Regulators of Bone Metabolism. Blood samples will be collected in the morning via venipuncture after an overnight fast (of ≥10 hours) and abstinence from physical activity for the previous 24 hours for later analyses of bone turnover and other biomarkers. Aliquots of plasma and serum will be stored at -70ºC in the Gerontology BioRepository directed by Dr. Nicklas until use in future assays.

D3-Creatine (D3Cr) muscle mass: Participants will be asked to ingest a small tablet containing 30 mg of D3-creatine, prepared by Wellspring Compounding Pharmacy (Berkeley, CA) to allow for a direct measure of muscle mass. 115 Participants will be given the dose at the SV2 and FV3 visits, and instructed to take the dose 3-6 days prior to their SV3 and FV4 visits (respectively) where they will be asked to provide a fasted urine sample. Urine will be collected either in a specimen cup and aliquoted, or will be collected directly on a filter paper strip, which will be frozen until shipment to the central lab at the University of California, Berkeley for detection of labeled and unlabeled creatinine by LC/MS.

Descriptive Data, Potential Covariates, and Adverse Events. Covariate assessment will occur at the frequency described in **Table 3**. Briefly, baseline demographic data will be recorded based on participant self-report (at baseline only). Medical information on prior and existing co-morbidities and hospitalizations will also be ascertained by self-report. Collectively, this information will be used to assess 10 year major osteoporotic and hip fracture risk using the FRAX tool.[104] We will also record medication use by asking participants to bring in all medications (including nutritional supplements). Height without shoes will be measured to the nearest 0.1 centimeter using a stadiometer and body mass will be measured to the nearest 0.1 kilogram using a calibrated and certified balance beam scale. Height and body mass will be used to calculate body mass index (BMI). Waist circumference will be measured to the nearest 0.1 cm with a Gulick-II spring-retractable steel tape. Resting metabolic rate (RMR) will be measured in the morning after an overnight fast by indirect calorimetry. Upon arrival, participants are asked to lie quietly for 15-20 minutes before testing. Measurement of oxygen consumption and carbon dioxide production are collected continuously for at least 30 minutes and RMR is

calculated using the Weir equation. Accelerometry will be used to objectively assess physical activity over a seven day period at baseline, 6 and 12 months using a triaxial accelerometer and inclinometer.[105] Finally, questionnaires assessing fatigue/fatigability[106], pain[107], physical activity[108], and intervention adherence self-efficacy (with questionnaire specific to INVEST modeled on prior work[109]) will be assessed. If covariates are found to be unbalanced between groups, and related to study outcomes, these variables will be included as covariates in exploratory, secondary statistical analyses. Lastly, adverse events will be assessed and recorded by asking participants to complete a bi-monthly health status questionnaire. Spontaneously reported adverse events will also be collected and reported by study staff.

Data and Statistical Analyses

Data Management. Data will be collected on forms at the clinic site and transferred to a secure electronic database. Our web-based management system will assure integrity and validity. Dynamic reports and statistical analyses will monitor quality. A participant-based inventory system will track recruitment, retention, adherence, and missing data from entry through exit, close-out, and lock-down of final datasets.

Statistical Analyses. We will use intention-to-treat principles in full accordance with CONSORT guidelines. We will monitor serious adverse events regularly to maintain up-to-date safety information for reporting to the DSMB. All data will undergo range checks at the time of data entry and will be examined monthly by histograms and bivariate scatterplots to check for inconsistencies, unusual data needing further verification, and outliers. Plots of longitudinal observations will be used to inspect for unusual changes requiring verification against source documentation. Regression diagnostics and exploratory analyses will be performed to find appropriate transformations of variables if needed. Order of priority in choosing a transformation will be to satisfy: 1) linearity, 2) homogeneity, and 3) normality assumptions. We will attempt to identify baseline covariates that predict attrition and compliance; and if such covariates can be identified, the analyses may need to incorporate stratification by these factors to decrease bias. In addition, following the recommendations of the 2010 National Academy of Sciences report,[110] if the outcome is found to be missing at random, we will attempt to identify baseline covariates that predict attrition and use these covariates to impute missing data based on multiple imputation. Sensitivity analyses, using methods like pattern mixture models, will be carried out to explore the effect of missing outcomes on inference (if found to be not at random). Additionally, we will conduct sensitivity analyses of efficacy taking into account treatment compliance as a binary (compliers defined as >80% of prescribed vest wear time, >80% resistance training attendance, >80% of weight loss target) or continuous variable (proportion of treatment received).

Sample Size and Power. A total sample size of 150 (n=50 per group) is optimal to test hypotheses for the primary study aims. Using co-primary hypotheses demands use of a conservative Bonferroni correction of dividing the Type I error rate (α) in half for each aim to determine statistical significance.

Aim 1, Hypothesis 1. Based on our preliminary data demonstrating effectiveness of the WL+VEST intervention in preserving total hip aBMD, as well as effect size estimates generated from an 18 month trial of WL versus WL+RT on total hip vBMD, we anticipate that the WL+VEST group will decrease 3% from a baseline total hip vBMD value of 0.300 g/cm^3 over 12 months compared to 5.5-6.0% decrease in the WL alone group, leading to a difference range of 2.5-3.0%. In **Table 4**, we observe that assuming 12 month group differences in total hip vBMD between 0.0075 and 0.009 g/cm^3, we have at least 85% power to detect a statistically significant difference for the primary outcome assuming common group standard deviations of 0.010 g/cm^3 and using a 2-sided t-test under differing retention scenarios over 12 months at a 0.025 level of

Table 4. Power for Aim 1, Hypothesis 1.

12 Month Δ Total Hip vBMD g/cm^3: WL+VEST (Δ%)	12 Month Δ Total Hip vBMD g/cm^3: WL (Δ%)	12 Month Attrition		
		15%	13%	10%
		Power (%)*		
-0.009 (-3.0%)	-0.0165 (-5.5%)	87.7	88.6	89.7
-0.009 (-3.0%)	-0.018 (-6.0%)	96.7	97.1	97.5

*Assumes n=50/group and unadjusted SD=0.010 g/cm^3 and α=0.025 under 2-sided alternative hypothesis.

significance. *Although we do not have pilot data specifically assessing total hip trabecular vBMD (only total hip vBMD, which is an integral measure of the trabecular and cortical regions), the two are highly correlated, with prior studies showing enhanced responsivity of the trabecular region.*[111,112] *Thus, using total hip vBMD for our power calculations ensures adequate power for total hip trabecular vBMD as the primary outcome measure.*

Aim 1, Hypothesis 2. We anticipate that the change in total hip trabecular vBMD among the WL+VEST will be noninferior to the change among WL+RT. The noninferiority margin for the difference in 12-month total hip vBMD changes is -4% of baseline or -0.012 g/cm^3, as used previously.[113] Assuming 12-month equivalence between WL+VEST and WL+RT (i.e., WL+VEST and WL+RT 12 month mean are each 0.291 g/cm^3), then a baseline sample size of 50/group provides >95% power to establish noninferiority of WL+VEST compared to WL+RT. This assumes 1) the same SD of change (0.010 g/cm^3) as in Hypothesis 1; 2) attrition rate is a conservative 15% (better retention provides higher power); and 3) the Type I error rate is 0.0125, based on the convention of using 0.025 for a one-sided test, which is further divided in half due to having co-primary hypotheses.[114] Alternately, using a smaller noninferiority bound provides a stricter standard for the trial objectives. Using a margin of -2.5% of baseline (-0.0075 g/cm^3) difference between WL+VEST vs WL+RT, we have 85% power to establish noninferiority assuming the same SD as above (0.010 g/cm^3), 15% attrition, and a 0.0125 Type I error rate.

Aim 2, Hypothesis 1. We note that the CT estimates were partially derived from DXA-measured aBMD loss under WL, which results in similar estimated BMD losses assumed between the two groups. We anticipate that the WL+VEST group will decrease 3% from a baseline total hip aBMD value of 1.027 g/cm^2 over 12 months compared to 5.5% decrease in the WL alone group, leading to a difference of 0.0257 g/cm^2, or 2.5% of baseline. Using this magnitude of difference, 50 participants/group will provide 90% power to detect a statistically significant difference for the primary outcome assuming common group standard deviation of change of 0.033 g/cm^2, based on a 2-sided t-test under 85% retention at 12 months using a 0.025 Type I Error rate.

Aim 2, Hypothesis 2. We anticipate that the change in total hip aBMD among the WL+VEST will be noninferior to the change among WL+RT. The noninferiority margin (i.e. difference at which a clinically meaningful increase of nonvertebral fracture risk may occur[115]) for the difference in 12 month total hip aBMD changes is -2.13% of baseline or -0.022 g/cm^2. Assuming equivalence between WL+VEST and WL+RT (i.e., WL+VEST and WL+RT 12 month mean are each 3% reduced from baseline (0.996 g/cm^2) as used in Hypothesis 1), then a baseline sample size of 50/group provides 80% power to establish noninferiority of WL+VEST compared to WL+RT such that the lower bound of the one-sided 0.9875 confidence bound of the group mean differences will be greater than a -0.022 g/cm^2 difference between WL+VEST minus WL+RT. This assumes 1) the same common group SD of change (0.033 g/cm^2) as in Hypothesis 1; 2) attrition rate is 15%; and 3) the Type I error rate is 0.0125, based on the convention of using 0.025 for a one-sided confidence interval, which is further divided in half due to having co-primary hypotheses.

Aim 2, All other outcomes. Due to the challenges of prespecifying noninferiority boundaries for exploratory outcomes, all other analyses for the second aim will focus on the three group comparisons of WL, WL+VEST, and WL+RT. Based on the same baseline sample size of 50/group (n=150 overall), we will have 80% power to detect a relatively modest effect sizes (variance of means/within-group variance) of 0.116 under the attrition rate of 15% assumed above, assuming 2-tailed F-test and a 0.05 Type I error rate.

Statistical Analysis Plan. The primary aim for comparisons of total hip trabecular vBMD will be tested using a mixed model fit using the change in total hip trabecular vBMD at 12 months versus the treatment effect indicator for each of the three groups, adjusted for visit (6 or 12 months), visit by treatment interaction, sex (to account for randomization strata) and baseline value. A contrast statement will test change in total hip trabecular vBMD at 12 months in WL versus WL+VEST, and a statistically significant difference will be

established at $p<0.025$. Next, the noninferiority of WL+VEST compared to WL+RT will be determined based on whether the lower bound of the one-sided confidence interval for the estimated 12 month treatment effect of WL+VEST versus WL+RT overlaps the -4.0% non-inferiority boundary for total hip trabecular vBMD. This will be performed using $\alpha=0.0125$ to account for co-primary hypotheses as well as for using a 1-sided alternative hypothesis. Tests for both co-primary hypotheses will be based on different contrast statements from the same statistical model. Secondarily, we will repeat these analyses with the short-term effect at 6 months of both interventions as an exploratory analysis. For Aim 2, we will repeat these analyses for the 12 month effect (and secondarily 6 month effect) using total hip aBMD assessed by DXA as the outcome. Comparisons will be performed for both superiority of WL+VEST vs. WL as well as noninferiority of WL+VEST vs WL+RT based on a 2.13% noninferiority margin using the methods described above. Analytic models for all other Aim 2 outcomes will mirror the model used in the primary aim, with the important exception that comparisons will focus on statistical comparisons of group mean differences. All outcomes are assessed at 6 and 12 months, and while both visit time points will be estimated, the 12 month treatment effect will be considered of primary interest. Treatment effects for changes in outcome variables are compared using a mixed model fit with treatment group, visit, and treatment by visit interaction, adjusted for sex and baseline values of the outcome. Tests will be performed using contrast statements at 12 months (primarily) and 6 months (secondarily), and we will use the partial F-test $p<0.05$ for significance. Significant comparisons for secondary outcomes will further use comparisons of all three groups at each visit using $p<0.0167$ for pairwise significance. Lastly, we intend to explore the associations between 6 and 12 month change in vBMD or aBMD with change in secondary outcome measures (i.e., DXA and CT-acquired body composition, D3Cr muscle mass, biomarkers of bone turnover, bone regulating hormones/cytokines) using linear regression methods and adjusted for sex and treatment group, to explore potential mechanisms underlying the treatment effect. Secondary comparisons will not be adjusted for multiplicity other than for pairwise group comparisons. Statistical analyses will be conducted using SAS v9.4 (SAS Institute, Cary, NC) and R software.

<u>Statistical Analysis and Power Considerations for Limb Loading Sub-Study:</u>

Limb Loading Analysis**:** Data from the insoles will be downloaded from the smartphone and imported into MATLAB (Mathworks Inc, Natick, MA, USA). Limb loading outcomes will include peak impact force, loading rate, impulse, and cumulative loading. Peak impact force will be calculated as the largest peak in the first 25% of all foot contacts. Loading rate will be calculated as the peak impact force divided by the time to reach the peak impact force from initial foot contact (>10N). Impulse for each foot contact (e.g. step, exercise repetition) is the area under the force curve. Cumulative loading will be defined as the total impulse from all recorded foot contacts. These outcomes will be determined for the right limb and will be averaged for each day of use with the daily average for each metric used for data analysis.

Femoral Stress and Strain Analysis: The baseline subject-specific proximal femur models developed in the parent study will be utilized for the proposed pilot to conduct additional FE simulations that prescribe the insole daily limb loads to estimate *in vivo* femoral stress and strain. The "peak impact force daily average" and "loading rate daily average" measured by the insoles will be applied to the femoral head of the FE model by prescribing a "force vs. time" curve that ramps up to the peak impact force in the time from initial foot contact (time=peak impact force divided by loading rate). The maximum femoral stress and femoral strain attained during the simulation will be output from the proximal femur, along with the location of those elements (e.g. femoral neck, trochanter, shaft). A strain of approximately 0.04 – 0.10% is estimated to be the minimum effective strain required to prevent bone loss. Strain values derived from our simulation will be compared to these values to determine if daily limb loading imposes adequate strain for maintenance of BMD.

<u>Feasibility Analysis</u>**:** Feasibility of wearing force-sensing insoles will be assessed by recruitment, adherence, and satisfaction. Recruitment rates and adherence to wearing the insoles over 4 days will be recorded, and overall satisfaction will be measured with an overall satisfaction questionnaire.

To evaluate limb loading, an analysis of covariance (ANCOVA) to test for mean differences between groups in daily loading metrics (cumulative loading, peak force, loading rate, impulse). To evaluate femoral stress and strain, an ANCOVA will also be used with femoral mechanical stress and strain as the dependent variable. In the event of significant differences, post-hoc independent two-sample t-tests using a Bonferroni adjusted alpha = 0.025 will further be tested. Fifteen participants per group provides 80% power to detect a moderate effect size (Variance between/Variance within) of 0.40 using a 0.05 Type I error rate and 87% retention at 6 months. Cohen's *d* effect sizes will be calculated to further contextualize group differences and inform future sample size calculations. Pearson product moment will also to explore associations between daily limb loading metrics, change in areal and volumetric BMD, and changes in physical function separately by group and across all groups combined.

Human Subjects Protection

Subject Recruitment Methods: We will recruit individuals using community-based recruitment strategies including newspaper ads and mass mailings. We will also advertise in the Sticht Center recruitment newsletter (Volunteers in Touch with an Active Lifestyle: VITAL), utilize WakeOne recruitment letters, and participate in community outreach events.

Informed Consent: Written informed consent will be obtained from each subject. The informed consent process will follow the procedures of the WFSM Institutional Review Board. The study interviewers will explain the purpose, methods and extent of the study to prospective participants. The potential participant is asked to read the informed consent form and ask questions. The form is written in simple easy to understand language. We require study staff to review all of the key aspects of the study verbally with the potential participants. Staff is provided with a structured checklist for this purpose. Staff is then required to question potential participants to ascertain whether s/he has understood the information. Potential participants who are illiterate or have impaired vision must have the consent read to them, followed by review of the checklist, opportunity for questions, and discussion. This process will take place in a quiet, private room. A copy of the signed and dated consent form will be given to participants, and the original document will be placed in subjects' individual study files, which will be stored in a secure location. In compliance with the Health Insurance Portability and Accountability Act (HIPAA) and the Standards for Privacy of Individually Identifiable Health Information of the Department of Health and Human Services, we will access personal health information only after obtaining informed consent.

Potential Risks: There are inherent potential risks to human subjects who participate in any research study and the potential risks to study participants in this study are listed below. Any injuries or illnesses (severe adverse events) during the course of a participant's enrollment in the study are monitored regularly as described below in the Data Safety Monitoring Plan.

Intervention risks:

1) Risks of weight loss at any age include the concomitant loss of fat-free (*i.e.*, bone and muscle) tissue along with fat mass loss; and in older adults, this may exacerbate age-related risk of osteoporotic fracture. Because the individual risk of osteoporotic fracture is highest in those who are underweight, this risk is mitigated by excluding individuals who are non-obese and less likely to benefit from weight loss and excluding those who are already diagnosed with osteoporosis (identified on any of the following: participant self-reported medical history, medication use, or baseline DXA scan). Procedures to minimize loss of bone during the weight loss intervention include prescription of a caloric deficit that does not result in excessively rapid weight loss (e.g., >2 lb/week), recommendation to engage in daily light- to moderate-intensity walking, in accordance with national physical activity guidelines, and incorporating meal replacements and meal plans that provide adequate protein, calcium, and vitamin D intake. In addition, because the vast majority of fractures occur subsequent to a fall, intervention staff

will hold fall safety discussions with all participants during group sessions, including distribution of written fall prevention resource materials. Fall safety instruction will occur during the first week of intervention and will be reiterated during the first week of the intervention transition phase (month 7).

2) OPTAVIA Fuelings utilize a variety of protein sources, including soy protein. Because of this, we will be alert to possible soy allergies and those taking thyroid medications. Most commonly, a soy allergy presents with mild signs and symptoms that may include itching, skin breakouts, or redness, nasal congestion or digestive issues. Additionally, consuming OPTAVIA products containing soy protein may decrease the body's absorption of thyroid medications, such as Synthroid or Levothyroxine. Those with a mild soy allergy or intolerance, or those taking thyroid medications, will be informed of the soy ingredients in the OPTAVIA Fuelings, and will be offered soy protein-free Fuelings. Participants taking thyroid medications will also be instructed to avoid eating any Fuelings containing soy protein within one to three hours before and after taking thyroid medication. Soy protein only needs to be avoided around the time thyroid medications are taken.

3) The risks of intervention-related problems with wearing the weighted vests may include: 1) an increased fall risk and 2) an exacerbation of prior conditions related to knee/back/shoulder pain. We exclude potential participants who report knee/back/shoulder pain with prescription medication use during the initial phone screen. Participants will also be advised to not wear the weighted vest during strenuous activities. Additionally, at the outset of all group sessions, participants will be queried regarding problems or health symptoms they may be experiencing with the vest. The interventionist will record the presence or absence of such symptoms and determine whether or not the contact should be handled emergently or, in consultation with the study physician, by a scheduled appointment with the primary care provider.

4) The risks of the structured resistance exercise intervention may include: 1) musculoskeletal complications and muscle soreness in the early phases of the intervention, and 2), an increased fall risk. These risks are minimized since all center-based exercise sessions will be supervised by trained exercise technicians who will instruct participants in proper balance safety and footwear to use. Procedures to minimize musculoskeletal and cardiac injury and muscle soreness include warm-up and cool-down activities that include large muscle movements and stretching. Participants will begin with an easier exercise stimulus and will gradually increase in intensity over the first few months. Exercise physiologists, trained in cardiac life support, will supervise all center-based exercise sessions and practice codes are conducted quarterly.

Assessment risks: Health risks for each of the assessment procedures may include:

1) Exposure to radiation from the DXA and CT scans. The amount of radiation that participants will receive from these procedures is equivalent to a uniform whole body dose of 1866 millirem. This is equal to 6.22 times the average yearly radiation exposure from background radiation (300 mRem). If a repeat CT scan is needed, participants will receive approximately 975 millirem of additional radiation, which would increase the uniform whole body dose of radiation to 2841 millitem. This is equal to 9.47 times the average yearly radiation exposure from background radiation (300 mRem). The potential long-term risk from these radiation doses is uncertain, but these doses are not associated with any definite adverse effects. Thus the risk to participants, if any, is estimated to be slight.

2) A small risk of injury during the muscle strength and function tests, such as muscle strains or pulls, falls, or joint injury. However, these tests have been performed in large study populations with no significant adverse events reported. Risks will be minimized by having experienced/trained staff conducting these

assessments. A warm-up and range of motion practice will be conducted before testing. In addition, if a participant reports pain, dizziness, lightheadedness or other medical problem during the test, the test will be terminated. During the walking trials with the force-sensing insoles, there is a small risk of fatigue, loss of balance, or falls. Breaks will be provided to participants to minimize fatigue, and participants will be spotted during the walking tests to prevent falls and injury.

3) Slight discomfort, bruising, and/or infection at the sight of puncture for blood drawing, but blood will be drawn only be trained and experienced phlebotomists who will minimize the discomfort as much as possible.

4) Risks associated with wearing the activity monitors are minimal, but may include minor skin irritations. Participants will be instructed to note these on their log and if it becomes bothersome to remove the device and call staff for further instructions.

Safety measures during the interventions: For the dietary intervention, all study participants are monitored for compliance to a safe level of caloric deficit. Since the degree of bone and muscle loss is accelerated by the rate of weight loss, all participants will be weighed weekly and if weight is being lost at a rate greater than 0.9 kg/wk (~2 lb/wk), adjustments may be made to the meal plan to increase the level of calories recommended to slow down this excess rate of weight loss. As stated previously, at the outset of all group sessions, participants will be queried regarding problems or health symptoms they may be experiencing with the vest. The interventionist will record the presence or absence of such symptoms and determine whether or not the problem requires medical attention and, if so, to determine whether or not the contact should be handled emergently or, in consultation with the study physician, by a scheduled appointment with the primary care provider. Supervised RT sessions are conducted at a central location and are supervised by trained interventionists who monitor potential adverse experiences and symptoms. On-site staff trained in advanced cardiac life support are available to deal with medical emergencies. Also, institutional and community EMS services will be activated if needed. Procedures to minimize musculoskeletal discomfort to the exercise include teaching proper warm-up and cool down activities and providing instruction on correct exercise techniques and fall safety. If for any reason the participant reports a fall or other injury, or chest pain, shortness of breath, or dizziness, during a supervised RT session, they will be referred to their doctor, or study staff will notify the participant's doctor or other health care provider with their permission. If at any point during a RT session, participants develop chest pain, shortness of breath, or dizziness, they will be instructed to stop exercise, and to contact the study staff if these symptoms persist or recur. We monitor blood pressure and heart rate before and after all supervised RT sessions. If an intervention-related injury or illness does occur, the study medical staff will be consulted according to the type of symptom reported. In some instances, it may be appropriate to reduce or to temporarily suspend the participant's weight loss, vest use, or RT goals. If the injury or symptoms do not resolve after an appropriate period of time, the participant will be referred to his/her primary care provider for further evaluation. The participant will be encouraged to follow the primary care provider's instructions regarding intervention compliance.

Safety measures during the assessments: All study assessments are conducted by trained and certified staff. Safety precautions are taken during all testing by applying standardized stopping criteria. If the participant reports pain, tightness or pressure in the chest, feeling faint, lightheaded or dizzy, or any other medical problems, the test will be stopped. When there are medically relevant findings, the participant will be told the cause for concern, and may be advised to consult his or her physician. If given permission by the participant, a letter will be sent to her primary care physician stating the concern.

Confidentiality and Privacy: Confidentiality will be protected by collecting only information needed to assess study outcomes, minimizing to the fullest extent possible the collection of any information that could directly

identify subjects, and maintaining all study information in a secure manner. To help ensure subject privacy and confidentiality, only a unique study identifier will appear on the data collection form. Any collected patient identifying information corresponding to the unique study identifier will be maintained on a separate master log. The master log will be kept secure, with access limited to designated study personnel and Medifast Scientific and Clinical Affairs personnel. Following data collection subject identifying information will be destroyed at the earliest opportunity, consistent with data validation and study design, producing an anonymous analytical data set. Data access will be limited to study staff. Data and records will be kept locked and secured in the Department of Health and Exercise Science, WFU and WFSM, with any computer data password protected. No reference to any individual participant will appear in reports, presentations, or publications that may arise from the study. Limb loading data will be saved onto the smartphone with a unique ID number. Following each visit, data from the smartphone will be downloaded onto a password protected computer. Data on the smartphone will be immediately deleted.

Data and Safety Monitoring: The principal investigator will be responsible for the overall monitoring of the data and safety of study participants. The principal investigator will be assisted by other study staff, including the study physician.

Reporting of Unanticipated Problems, Adverse Events or Deviations: Any unanticipated problems, serious and unexpected adverse events, deviations or protocol changes will be promptly reported by the principal investigator or designated member of the research team to the IRB and sponsor or appropriate government agency if appropriate.

Use of biological samples by other investigators: Biological samples may be used by investigators other than the investigators of the current study. The use will be limited to non-commercial purposes. The names and other personal identifiers of the study participants will not be sent to any recipients of the blood samples.

Storage and disposal of biological material: Tissue samples will be stored at Wake Forest University Medical Center indefinitely after the end of the trial. Biological specimens will be stored in locked alarmed ultra-low freezers located in a locked room, and will be destroyed when they are no longer needed. The lab coordinator and the PI have access to the keys of the freezers. All the specimens will have numerical study IDs with no personal identifiers of the participants. These are stored under the Pepper Center Tissue Repository (IRB#1219).

BIBLIOGRAPHY AND REFERENCES CITED

1. Arterburn DE, Crane PK, Sullivan SD. The coming epidemic of obesity in elderly Americans. J Am Geriatr Soc. 2004 Nov;52(11):1907–1912. PMID: 15507070

2. Eiben G, Dey DK, Rothenberg E, Steen B, Björkelund C, Bengtsson C, Lissner L. Obesity in 70-year-old Swedes: secular changes over 30 years. Int J Obes. 2005 Jul;29(7):810–817. PMID: 15917864

3. Stenholm S, Simonsick EM, Ferrucci L. Secular trends in body weight in older men born between 1877 and 1941: the Baltimore Longitudinal Study of Aging. J Gerontol A Biol Sci Med Sci. 2010 Jan;65(1):105–110. PMID: 19933750

4. Flegal KM, Carroll MD, Kit BK, Ogden CL. Prevalence of obesity and trends in the distribution of body mass index among US adults, 1999-2010. JAMA. 2012 Feb 1;307(5):491–497. PMID: 22253363

5. Ogden CL, Carroll MD, Fryar CD, Flegal KM. Prevalence of Obesity Among Adults and Youth: United States, 2011-2014. NCHS Data Brief. 2015 Nov;(219):1–8. PMID: 26633046

6. Houston DK, Nicklas BJ, Zizza CA. Weighty concerns: the growing prevalence of obesity among older adults. J Am Diet Assoc. 2009 Nov;109(11):1886–1895. PMID: 19857630.

7. Waters DL, Ward AL, Villareal DT. Weight loss in obese adults 65 years and older: a review of the controversy. Exp Gerontol. 2013 Oct;48(10):1054–1061. PMID: 23403042

8. Bales CW, Buhr G. Is obesity bad for older persons? A systematic review of the pros and cons of weight reduction in later life. J Am Med Dir Assoc. 2008 Jun;9(5):302–312. PMID: 18519110

9. Batsis JA, Gill LE, Masutani RK, Adachi-Mejia AM, Blunt HB, Bagley PJ, Lopez-Jimenez F, Bartels SJ. Weight loss interventions in older adults with obesity: a systematic review of randomized controlled trials since 2005. J Am Geriatr Soc. 2017 Feb;65(2):257–268. PMID: 27641543

10. Locher JL, Goldsby TU, Goss AM, Kilgore ML, Gower B, Ard JD. Calorie restriction in overweight older adults: Do benefits exceed potential risks? Exp Gerontol. 2016 Dec 15;86:4–13. PMID: 26994938

11. Ensrud KE, Ewing SK, Stone KL, Cauley JA, Bowman PJ, Cummings SR. Intentional and unintentional weight loss increase bone loss and hip fracture risk in older women. J Am Geriatr Soc. 2003 Dec;51(12):1740–1747. PMID: 14687352

12. Ensrud KE, Fullman RL, Barrett-Connor E, Cauley JA, Stefanick ML, Fink HA, Lewis CE, Orwoll E. Voluntary weight reduction in older men increases hip bone loss: the osteoporotic fractures in men study. J Clin Endocrinol Metab. 2005 Apr;90(4):1998–2004. PMID: 15671096

13. Dennison E, Eastell R, Fall CH, Kellingray S, Wood PJ, Cooper C. Determinants of bone loss in elderly men and women: a prospective population-based study. Osteoporos Int. 1999;10(5):384–391. PMID: 10591836

14. Hannan MT, Felson DT, Dawson-Hughes B, Tucker KL, Cupples LA, Wilson PW, Kiel DP. Risk factors for longitudinal bone loss in elderly men and women: the Framingham Osteoporosis Study. J Bone Min Res. 2000 Apr;15(4):710–720. PMID: 10780863

15. Knoke JD, Barrett-Connor E. Weight loss: a determinant of hip bone loss in older men and women. The Rancho Bernardo Study. Am J Epidemiol. 2003 Dec 15;158(12):1132–1138. PMID: 14652297

16. Shapses SA, Sukumar D. Bone metabolism in obesity and weight loss. Annu Rev Nutr. 2012 Aug 21;32(1):287–309. PMID: 22809104

17. Zibellini J, Seimon RV, Lee CM, Gibson AA, Hsu MS, Shapses SA, Nguyen TV, Sainsbury A. Does diet-induced weight loss lead to bone loss in overweight or obese adults? A systematic review and meta-analysis of clinical trials. J Bone Miner Res. 2015 Dec 1;30(12):2168–2178. PMID: 26012544

18. Villareal DT, Fontana L, Das SK, Redman L, Smith SR, Saltzman E, Bales C, Rochon J, Pieper C, Huang M, Lewis M, Schwartz AV, CALERIE Study Group. Effect of two-year caloric restriction on bone metabolism and bone mineral density in non-obese younger adults: a randomized clinical trial. J Bone Miner Res. 2016 Jan;31(1):40–51. PMID: 26332798

19. Iwaniec UT, Turner RT. Influence of body weight on bone mass, architecture and turnover. J Endocrinol. 2016 Sep;230(3):R115-130. PMID: 27352896

20. Schwartz AV, Johnson KC, Kahn SE, Shepherd JA, Nevitt MC, Peters AL, Walkup MP, Hodges A, Williams CC, Bray GA, Look AHEAD Research Group. Effect of 1 year of an intentional weight loss intervention on bone mineral density in type 2 diabetes: results from the Look AHEAD randomized trial. J Bone Miner Res. 2012 Mar;27(3):619–627. PMCID: PMC3410035

21. Lipkin EW, Schwartz AV, Anderson AM, Davis C, Johnson KC, Gregg EW, Bray GA, Berkowitz R, Peters AL, Hodges A, Lewis C, Kahn SE, Look AHEAD Research Group. The Look AHEAD Trial: bone loss at 4-year follow-up in type 2 diabetes. Diabetes Care. 2014 Oct;37(10):2822–2829. PMCID: PMC4170123

22. Nguyen TV, Sambrook PN, Eisman JA. Bone loss, physical activity, and weight change in elderly women: the Dubbo Osteoporosis Epidemiology Study. J Bone Miner Res. 1998 Sep;13(9):1458–1467. PMID: 9738519

23. Lang TF, Sigurdsson S, Karlsdottir G, Oskarsdottir D, Sigmarsdottir A, Chengshi J, Kornak J, Harris TB, Sigurdsson G, Jonsson BY, Siggeirsdottir K, Eiriksdottir G, Gudnason V, Keyak JH. Age-related loss of proximal femoral strength in elderly men and women: the Age Gene/Environment Susceptibility Study--Reykjavik. Bone. 2012 Mar;50(3):743–748. PMID: 22178403

24. Langlois JA, Visser M, Davidovic LS, Maggi S, Li G, Harris TB. Hip fracture risk in older white men is associated with change in body weight from age 50 years to old age. Arch Intern Med. 1998 May 11;158(9):990–996. PMID: 9588432

25. Ensrud KE, Cauley J, Lipschutz R, Cummings SR. Weight change and fractures in older women. Study of Osteoporotic Fractures Research Group. Arch Intern Med. 1997 Apr 28;157(8):857–863. PMID: 9129545

26. Crandall CJ, Yildiz VO, Wactawski-Wende J, Johnson KC, Chen Z, Going SB, Wright NC, Cauley JA. Postmenopausal weight change and incidence of fracture: post hoc findings from Women's Health Initiative Observational Study and Clinical Trials. BMJ. 2015;350:h25. PMID: 25627698

27. Johnson KC, Bray GA, Cheskin LJ, Clark JM, Egan CM, Foreyt JP, Garcia KR, Glasser S, Greenway FL, Gregg EW, Hazuda HP, Hergenroeder A, Hill JO, Horton ES, Jakicic JM, Jeffery RW, Kahn SE, Knowler WC, Lewis CE, Miller M, Montez MG, Nathan DM, Patricio JL, Peters AL, Pi-Sunyer X, Pownall HJ, Reboussin D, Redmon JB, Steinberg H, Wadden TA, Wagenknecht LE, Wing RR, Womack CR, Yanovski SZ, Zhang P, Schwartz AV, Look AHEAD Study Group. The effect of intentional weight loss on fracture

risk in persons with diabetes: results from the Look AHEAD randomized clinical trial. J Bone Miner Res. 2017 Nov;32(11):2278–2287. PMCID: PMC5685890

28. Lv Q-B, Fu X, Jin H-M, Xu H-C, Huang Z-Y, Xu H-Z, Chi Y-L, Wu A-M. The relationship between weight change and risk of hip fracture: meta-analysis of prospective studies. Sci Rep. 2015 Nov 2;5:16030. PMCID: PMC4629201

29. Cockerill W, Lunt M, Silman AJ, Cooper C, Lips P, Bhalla AK, Cannata JB, Eastell R, Felsenberg D, Gennari C, Johnell O, Kanis JA, Kiss C, Masaryk P, Naves M, Poor G, Raspe H, Reid DM, Reeve J, Stepan J, Todd C, Woolf AD, O’Neill TW. Health-related quality of life and radiographic vertebral fracture. Osteoporos Int. 2004 Feb;15(2):113–119. PMID: 14618303

30. Center JR, Nguyen TV, Schneider D, Sambrook PN, Eisman JA. Mortality after all major types of osteoporotic fracture in men and women: an observational study. Lancet. 1999 Mar 13;353(9156):878–882. PMID: 10093980

31. The Burden of Bone Disease. Bone Health Osteoporos Rep Surg Gen. Rockville, MD: Office of the Surgeon General (US); 2004.

32. Colón-Emeric CS, Saag KG. Osteoporotic fractures in older adults. Best Pract Res Clin Rheumatol. 2006 Aug;20(4):695–706. PMID: 16979533

33. Villalon KL, Gozansky WS, Van Pelt RE, Wolfe P, Jankowski CM, Schwartz RS, Kohrt WM. A losing battle: weight regain does not restore weight loss-induced bone loss in postmenopausal women. Obesity. 2011 Dec;19(12):2345–2350. PMID: 21852813

34. Avenell A, Richmond PR, Lean ME, Reid DM. Bone loss associated with a high fibre weight reduction diet in postmenopausal women. Eur J Clin Nutr. 1994 Aug;48(8):561–566. PMID: 7957001

35. Park HA, Lee JS, Kuller LH, Cauley JA. Effects of weight control during the menopausal transition on bone mineral density. J Clin Endocrinol Metab. 2007 Oct;92(10):3809–3815. PMID: 17635939

36. Von Thun NL, Sukumar D, Heymsfield SB, Shapses SA. Does bone loss begin after weight loss ends? Results 2 years after weight loss or regain in postmenopausal women. Menopause. 2014 May;21(5):501–508. PMCID: PMC5032655

37. Frost HM. Bone “mass” and the “mechanostat”: a proposal. Anat Rec. 1987 Sep;219(1):1–9. PMID: 3688455

38. Kohrt WM, Barry DW, Schwartz RS. Muscle Forces or Gravity: What Predominates Mechanical Loading on Bone? Med Sci Sports Exerc. 2009 Nov;41(11):2050–2055. PMCID: PMC3037021

39. Bolam KA, van Uffelen JG, Taaffe DR. The effect of physical exercise on bone density in middle-aged and older men: A systematic review. Osteoporos Int. 2013 Apr 4; PMID: 23552825

40. Bolam KA, Skinner TL, Jenkins DG, Galvão DA, Taaffe DR. The osteogenic effect of impact-loading and resistance exercise on bone mineral density in middle-aged and older men: a pilot study. Gerontology. 2015;62(1):22–32. PMID: 26226987

41. Martyn-St JM, Carroll S. High-intensity resistance training and postmenopausal bone loss: a meta-analysis. Osteoporos Int. 2006;17(8):1225–1240. PMID: 16823548

42. Ryan AS, Nicklas BJ, Dennis KE. Aerobic exercise maintains regional bone mineral density during weight loss in postmenopausal women. J Appl Physiol. 1998 Apr;84(4):1305–1310. PMID: 9516197

43. Villareal DT, Fontana L, Weiss EP, Racette SB, Steger-May K, Schechtman KB, Klein S, Holloszy JO. Bone mineral density response to caloric restriction-induced weight loss or exercise-induced weight loss: a randomized controlled trial. Arch Intern Med. 2006 Dec 11;166(22):2502–2510. PMID: 17159017

44. Daly RM, Dunstan DW, Owen N, Jolley D, Shaw JE, Zimmet PZ. Does high-intensity resistance training maintain bone mass during moderate weight loss in older overweight adults with type 2 diabetes? Osteoporos Int. 2005 Dec;16(12):1703–1712. PMID: 15937634

45. Shah K, Armamento-Villareal R, Parimi N, Chode S, Sinacore DR, Hilton TN, Napoli N, Qualls C, Villareal DT. Exercise training in obese older adults prevents increase in bone turnover and attenuates decrease in hip bone mineral density induced by weight loss despite decline in bone-active hormones. J Bone Min Res. 2011 Dec;26(12):2851–2859. PMID: 21786319

46. Hosny IA, Elghawabi HS, Younan WBF, Sabbour AA, Gobrial MAM. Beneficial impact of aerobic exercises on bone mineral density in obese premenopausal women under caloric restriction. Skeletal Radiol. 2012 Apr;41(4):423–427. PMID: 21604211

47. Beavers KM, Ambrosius WT, Rejeski WJ, Burdette JH, Walkup MP, Sheedy JL, Nesbit BA, Gaukstern JE, Nicklas BJ, Marsh AP. Effect of exercise type during intentional weight loss on body composition in older adults with obesity. Obesity. 2017 Nov;25(11):1823–1829. PMCID: PMC5678994

48. Courteix D, Valente-dos-Santos J, Ferry B, Lac G, Lesourd B, Chapier R, Naughton G, Marceau G, João Coelho-e-Silva M, Vinet A, Walther G, Obert P, Dutheil F. Multilevel Approach of a 1-Year Program of Dietary and Exercise Interventions on Bone Mineral Content and Density in Metabolic Syndrome--the RESOLVE Randomized Controlled Trial. PloS One. 2015;10(9):e0136491. PMID: 26376093

49. Physical Activity and Health: A Report of the Surgeon General | CDC [Internet]. [cited 2017 Jan 5]. Available from: https://www.cdc.gov/nccdphp/sgr/

50. Silver FH, Siperko LM. Mechanosensing and mechanochemical transduction: how is mechanical energy sensed and converted into chemical energy in an extracellular matrix? Crit Rev Biomed Eng. 2003;31(4):255–331. PMID: 15095950

51. Marden JH, Fescemyer HW, Saastamoinen M, MacFarland SP, Vera JC, Frilander MJ, Hanski I. Weight and nutrition affect pre-mRNA splicing of a muscle gene associated with performance, energetics and life history. J Exp Biol. 2008 Dec;211(Pt 23):3653–3660. PMID: 19011203

52. Schilder RJ, Kimball SR, Marden JH, Jefferson LS. Body weight-dependent troponin T alternative splicing is evolutionarily conserved from insects to mammals and is partially impaired in skeletal muscle of obese rats. J Exp Biol. 2011 May 1;214(Pt 9):1523–1532. PMID: 21490260

53. Greendale GA, Hirsch SH, Hahn TJ. The effect of a weighted vest on perceived health status and bone density in older persons. Qual Life Res Int J Qual Life Asp Treat Care Rehabil. 1993 Apr;2(2):141–152. PMID: 8518768

54. Shaw JM, Snow CM. Weighted vest exercise improves indices of fall risk in older women. J Gerontol A Biol Sci Med Sci. 1998 Jan;53(1):M53-58. PMID: 9467434

55. Snow CM, Shaw JM, Winters KM, Witzke KA. Long-term exercise using weighted vests prevents hip bone loss in postmenopausal women. J Gerontol A Biol Sci Med Sci. 2000 Sep;55(9):M489-491. PMID: 10995045

56. Bean J, Herman S, Kiely DK, Callahan D, Mizer K, Frontera WR, Fielding RA. Weighted stair climbing in mobility-limited older people: a pilot study. J Am Geriatr Soc. 2002 Apr;50(4):663–670. PMID: 11982666

57. Bean JF, Herman S, Kiely DK, Frey IC, Leveille SG, Fielding RA, Frontera WR. Increased Velocity Exercise Specific to Task (InVEST) training: a pilot study exploring effects on leg power, balance, and mobility in community-dwelling older women. J Am Geriatr Soc. 2004 May;52(5):799–804. PMID: 15086665

58. Klentrou P, Slack J, Roy B, Ladouceur M. Effects of exercise training with weighted vests on bone turnover and isokinetic strength in postmenopausal women. J Aging Phys Act. 2007 Jul;15(3):287–299. PMID: 17724395

59. Roghani T, Torkaman G, Movasseghe S, Hedayati M, Goosheh B, Bayat N. Effects of short-term aerobic exercise with and without external loading on bone metabolism and balance in postmenopausal women with osteoporosis. Rheumatol Int. 2013 Feb;33(2):291–298. PMID: 22441962

60. Jessup JV, Horne C, Vishen RK, Wheeler D. Effects of exercise on bone density, balance, and self-efficacy in older women. Biol Res Nurs. 2003 Jan;4(3):171–180. PMID: 12585781

61. Hakestad KA, Torstveit MK, Nordsletten L, Axelsson ÅC, Risberg MA. Exercises including weight vests and a patient education program for women with osteopenia: a feasibility study of the OsteoACTIVE rehabilitation program. J Orthop Sports Phys Ther. 2015 Feb;45(2):97–105, C1-4. PMID: 25579693

62. Kelleher JL, Beavers DP, Henderson RM, Yow D, Crotts C, Kiel J, Nicklas BJ, Beavers KM. Weighted Vest Use during Dietary Weight Loss on Bone Health in Older Adults with Obesity. J Osteoporos Phys Act. 2017;5(4). PMCID: PMC5788462

63. Engelke K, Lang T, Khosla S, Qin L, Zysset P, Leslie WD, Shepherd JA, Schousboe JT. Clinical use of quantitative computed tomography (qct) of the hip in the management of osteoporosis in adults: the 2015 ISCD official positions-part I. J Clin Densitom. 2015 Sep;18(3):338–358. PMID: 26277851

64. Zysset P, Qin L, Lang T, Khosla S, Leslie WD, Shepherd JA, Schousboe JT, Engelke K. Clinical use of quantitative computed tomography-based finite element analysis of the hip and spine in the management of osteoporosis in adults: the 2015 ISCD official positions-part II. J Clin Densitom. 2015 Sep;18(3):359–392. PMID: 26277852

65. Jensen MD, Ryan DH, Apovian CM, Ard JD, Comuzzie AG, Donato KA, Hu FB, Hubbard VS, Jakicic JM, Kushner RF, Loria CM, Millen BE, Nonas CA, Pi-Sunyer FX, Stevens J, Stevens VJ, Wadden TA, Wolfe BM, Yanovski SZ. 2013 AHA/ACC/TOS guideline for the management of overweight and obesity in adults: a report of the American College of Cardiology/American Heart Association task force on practice guidelines and The Obesity Society. Circulation. 2013 Nov 12; PMID: 24222017

66. Hobson J. The Montreal Cognitive Assessment (MoCA). Occup Med Oxf Engl. 2015 Dec;65(9):764–765. PMID: 26644445

67. Beavers KM, Gordon MM, Easter L, Beavers DP, Hairston KG, Nicklas BJ, Vitolins MZ. Effect of protein source during weight loss on body composition, cardiometabolic risk and physical performance in abdominally obese, older adults: a pilot feeding study. J Nutr Health Aging. 2015 Jan;19(1):87–95. PMID: 25560821

68. Beavers KM, Nesbit BA, Kiel JR, Sheedy JL, Arterburn LM, Collins AE, Ford SA, Henderson RM, Coleman CD, Beavers DP. Effect of an Energy-Restricted, Nutritionally Complete, Higher Protein Meal Plan on Body Composition and Mobility in Older Adults With Obesity: A Randomized Controlled Trial. J Gerontol A Biol Sci Med Sci. 2019 May 16;74(6):929–935. PMCID: PMC6521917

69. Normandin E, Yow D, Crotts C, Kiel J, Beavers KM, Nicklas BJ. Feasibility of Weighted Vest Use during a Dietary Weight Loss Intervention and Effects on Body Composition and Physical Function in Older Adults. J Frailty Aging. 2018;7(3):198–203. PMID: 30095153

70. Rejeski WJ, Brubaker PH, Goff DC Jr, Bearon LB, McClelland JW, Perri MG, Ambrosius WT. Translating weight loss and physical activity programs into the community to preserve mobility in older, obese adults in poor cardiovascular health. Arch Intern Med. 2011 May 23;171(10):880–886. PMID: 21263080

71. Rejeski WJ, Ambrosius WT, Burdette JH, Walkup MP, Marsh AP. Community weight loss to combat obesity and disability in at-risk older adults. J Gerontol A Biol Sci Med Sci. 2017 Jan 6; PMID: 28064148

72. Ryan DH, Espeland MA, Foster GD, Haffner SM, Hubbard VS, Johnson KC, Kahn SE, Knowler WC, Yanovski SZ, Look AHEAD Research Group. Look AHEAD (Action for Health in Diabetes): design and methods for a clinical trial of weight loss for the prevention of cardiovascular disease in type 2 diabetes. Control Clin Trials. 2003 Oct;24(5):610–628. PMID: 14500058

73. Jakicic JM, Clark K, Coleman E, Donnelly JE, Foreyt J, Melanson E, Volek J, Volpe SL. American College of Sports Medicine position stand. Appropriate intervention strategies for weight loss and prevention of weight regain for adults. Med Sci Sports Exerc. 2001 Dec;33(12):2145–2156. PMID: 11740312

74. Piercy KL, Troiano RP, Ballard RM, Carlson SA, Fulton JE, Galuska DA, George SM, Olson RD. The Physical Activity Guidelines for Americans. JAMA. 2018 20;320(19):2020–2028. PMID: 30418471

75. Morgan EF, Bayraktar HH, Keaveny TM. Trabecular bone modulus-density relationships depend on anatomic site. J Biomech. 2003 Jul;36(7):897–904. PMID: 12757797

76. Kopperdahl DL, Morgan EF, Keaveny TM. Quantitative computed tomography estimates of the mechanical properties of human vertebral trabecular bone. J Orthop Res Off Publ Orthop Res Soc. 2002 Jul;20(4):801–805. PMID: 12168670

77. Schileo E, Dall'ara E, Taddei F, Malandrino A, Schotkamp T, Baleani M, Viceconti M. An accurate estimation of bone density improves the accuracy of subject-specific finite element models. J Biomech. 2008 Aug 7;41(11):2483–2491. PMID: 18606417

78. Beavers DP, Beavers KM, Loeser RF, Walton NR, Lyles MF, Nicklas BJ, Shapses SA, Newman JJ, Messier SP. The independent and combined effects of intensive weight loss and exercise training on bone mineral density in overweight and obese older adults with osteoarthritis. Osteoarthritis Cartilage. 2014 Jun;22(6):726–733. PMID: 24742955

79. Beavers KM, Beavers DP, Martin SB, Marsh AP, Lyles MF, Lenchik L, Shapses SA, Nicklas BJ. Change in bone mineral density during weight loss with resistance versus aerobic exercise training in older adults. J Gerontol A Biol Sci Med Sci. 2017 Oct 12;72(11):1582–1585. PMCID: PMC5861903

80. Nicklas BJ, Chmelo E, Delbono O, Carr JJ, Lyles MF, Marsh AP. Effects of resistance training with and without caloric restriction on physical function and mobility in overweight and obese older adults: a randomized controlled trial. Am J Clin Nutr. 2015 May;101(5):991–999. PMID: 25762810

81. Nicklas BJ, Wang X, You T, Lyles MF, Demons J, Easter L, Berry MJ, Lenchik L, Carr JJ. Effect of exercise intensity on abdominal fat loss during calorie restriction in overweight and obese postmenopausal women: a randomized, controlled trial. Am J Clin Nutr. 2009 Apr;89(4):1043–1052. PMID: 19211823

82. Beavers KM, Ambrosius WT, Nicklas BJ, Rejeski WJ. Independent and combined effects of physical activity and weight loss on inflammatory biomarkers in overweight and obese older adults. J Am Geriatr Soc. 2013 Jul;61(7):1089–1094. PMCID: PMC3714323

83. Miller GD, Nicklas BJ, Loeser RF. Inflammatory biomarkers and physical function in older, obese adults with knee pain and self-reported osteoarthritis after intensive weight-loss therapy. J Am Geriatr Soc. 2008 Apr;56(4):644–651. PMID: 18312558

84. Boutin RD, Bamrungchart S, Bateni CP, Beavers DP, Beavers KM, Meehan JP, Lenchik L. CT of patients with hip fracture: muscle size and attenuation help predict mortality. Am J Roentgenol. 2017 Mar 7;W1–W8. PMID: 28267356

85. Weaver AA, Beavers KM, Hightower RC, Lynch SK, Miller AN, Stitzel JD. Lumbar bone mineral density phantomless computed tomography measurements and correlation with age and fracture incidence. Traffic Inj Prev. 2015;16 Suppl 2:S153-160. PMID: 26436225

86. Beavers KM, Miller ME, Rejeski WJ, Nicklas BJ, Krichevsky SB, Kritchevsky SB. Fat mass loss predicts gain in physical function with intentional weight loss in older adults. J Gerontol A Biol Sci Med Sci. 2013 Jan;68(1):80–86. PMID: 22503993

87. Lillie EM, Urban JE, Lynch SK, Weaver AA, Stitzel JD. Evaluation of skull cortical thickness changes with age and sex from computed tomography scans. J Bone Miner Res. 2016 Feb;31(2):299–307. PMID: 26255873

88. Schoell SL, Weaver AA, Urban JE, Jones DA, Stitzel JD, Hwang E, Reed MP, Rupp JD, Hu J. Development and validation of an older occupant finite element model of a mid-sized male for investigation of age-related injury risk. Stapp Car Crash J. 2015 Nov;59:359–383. PMID: 26660751

89. Treece GM, Gee AH, Mayhew PM, Poole KES. High resolution cortical bone thickness measurement from clinical CT data. Med Image Anal. 2010 Jun;14(3):276–290. PMID: 20163980

90. Treece GM, Poole KES, Gee AH. Imaging the femoral cortex: thickness, density and mass from clinical CT. Med Image Anal. 2012 Jul;16(5):952–965. PMID: 22465079

91. Schoell SL, Weaver AA, Vavalle NA, Stitzel JD. Age- and sex-specific thorax finite element model development and simulation. Traffic Inj Prev. 2015;16 Suppl 1:S57-65. PMID: 26027976

92. Vavalle NA, Schoell SL, Weaver AA, Stitzel JD, Gayzik FS. Application of radial basis function methods in the development of a 95th percentile male seated FEA model. Stapp Car Crash J. 2014 Nov;58:361–384. PMID: 26192960

93. Keyak JH, Rossi SA, Jones KA, Skinner HB. Prediction of femoral fracture load using automated finite element modeling. J Biomech. 1998 Feb;31(2):125–133. PMID: 9593205

94. Crawford RP, Cann CE, Keaveny TM. Finite element models predict in vitro vertebral body compressive strength better than quantitative computed tomography. Bone. 2003 Oct;33(4):744–750. PMID: 14555280

95. Weaver AA, Nguyen CM, Schoell SL, Maldjian JA, Stitzel JD. Image segmentation and registration algorithm to collect thoracic skeleton semilandmarks for characterization of age and sex-based thoracic morphology variation. Comput Biol Med. 2015 Dec 1;67:41–48. PMID: 26496701

96. Duque G. Bone and fat connection in aging bone. Curr Opin Rheumatol. 2008 Jul;20(4):429–434. PMID: 18525356

97. Tagliaferri C, Wittrant Y, Davicco M-J, Walrand S, Coxam V. Muscle and bone, two interconnected tissues. Ageing Res Rev. 2015 May;21:55–70. PMID: 25804855

98. Simonsick EM, Montgomery PS, Newman AB, Bauer DC, Harris T. Measuring fitness in healthy older adults: the Health ABC Long Distance Corridor Walk. J Am Geriatr Soc. 2001 Nov;49(11):1544–1548. PMID: 11890597

99. Pahor M, Guralnik JM, Ambrosius WT, Blair S, Bonds DE, Church TS, Espeland MA, Fielding RA, Gill TM, Groessl EJ, King AC, Kritchevsky SB, Manini TM, McDermott MM, Miller ME, Newman AB, Rejeski WJ, Sink KM, Williamson JD, investigators L study. Effect of structured physical activity on prevention of major mobility disability in older adults: the LIFE study randomized clinical trial. JAMA. 2014 Jun 18;311(23):2387–96. PMID: 24866862

100. Rolland YM, Cesari M, Miller ME, Penninx BW, Atkinson HH, Pahor M. Reliability of the 400-m usual-pace walk test as an assessment of mobility limitation in older adults. J Am Geriatr Soc. 2004 Jun;52(6):972–976. PMID: 15161464

101. Chang M, Cohen-Mansfield J, Ferrucci L, Leveille S, Volpato S, de Rekeneire N, Guralnik JM. Incidence of loss of ability to walk 400 meters in a functionally limited older population. J Am Geriatr Soc. 2004 Dec;52(12):2094–8. PMID: 15571549

102. Simonsick EM, Newman AB, Nevitt MC, Kritchevsky SB, Ferrucci L, Guralnik JM, Harris T. Measuring higher level physical function in well-functioning older adults: expanding familiar approaches in the Health ABC study. JGerontolA BiolSciMedSci. 2001 Oct;56(10):M644–M649.

103. Podsiadlo D, Richardson S. The timed “Up & Go”: a test of basic functional mobility for frail elderly persons. J Am Geriatr Soc. 1991 Feb;39(2):142–8. PMID: 1991946

104. Watts NB, Lewiecki EM, Miller PD, Baim S. National Osteoporosis Foundation 2008 clinician’s guide to prevention and treatment of osteoporosis and the world health organization fracture risk assessment tool (FRAX): what they mean to the bone densitometrist and bone technologist. J Clin Densitom. 2008 Dec;11(4):473–477. PMID: 18562228

105. Klenk J, Büchele G, Lindemann U, Kaufmann S, Peter R, Laszlo R, Kobel S, Rothenbacher D. Concurrent Validity of activPAL and activPAL3 Accelerometers in Older Adults. J Aging Phys Act. 2016;24(3):444–450. PMID: 26751290

106. Glynn NW, Santanasto AJ, Simonsick EM, Boudreau RM, Beach SR, Schulz R, Newman AB. The Pittsburgh Fatigability scale for older adults: development and validation. J Am Geriatr Soc. 2015 Jan;63(1):130–135. PMCID: PMC4971882

107. Revicki DA, Chen W-H, Harnam N, Cook KF, Amtmann D, Callahan LF, Jensen MP, Keefe FJ. Development and Psychometric Analysis of the PROMIS Pain Behavior Item Bank. Pain. 2009 Nov;146(1–2):158–169. PMCID: PMC2775487

108. Stewart AL, Mills KM, King AC, Haskell WL, Gillis D, Ritter PL. CHAMPS physical activity questionnaire for older adults: outcomes for interventions. Med Sci Sports Exerc. 2001 Jul;33(7):1126–1141. PMID: 11445760

109. McAuley E. Self-efficacy and the maintenance of exercise participation in older adults. J Behav Med. 1993 Feb;16(1):103–113. PMID: 8433355

110. The prevention and treatment of missing data in clinical trials. Washington (DC): National Academies Press (US); 2010. PMID: 24983040

111. Lang TF, Guglielmi G, van Kuijk C, De Serio A, Cammisa M, Genant HK. Measurement of bone mineral density at the spine and proximal femur by volumetric quantitative computed tomography and dual-energy x-ray absorptiometry in elderly women with and without vertebral fractures. Bone. 2002 Jan 1;30(1):247–250.

112. Genant HK, Libanati C, Engelke K, Zanchetta JR, Høiseth A, Yuen CK, Stonkus S, Bolognese MA, Franek E, Fuerst T, Radcliffe H-S, McClung MR. Improvements in hip trabecular, subcortical, and cortical density and mass in postmenopausal women with osteoporosis treated with denosumab. Bone. 2013 Oct 1;56(2):482–488.

113. Cheung AM, Tile L, Cardew S, Pruthi S, Robbins J, Tomlinson G, Kapral MK, Khosla S, Majumdar S, Erlandson M, Scher J, Hu H, Demaras A, Lickley L, Bordeleau L, Elser C, Ingle J, Richardson H, Goss PE. Bone density and structure in healthy postmenopausal women treated with exemestane for the primary prevention of breast cancer: a nested substudy of the MAP.3 randomised controlled trial. Lancet Oncol. 2012 Mar 1;13(3):275–284.

114. Chow S-C, Wang H, Shao J. Sample Size Calculations in Clinical Research, Second Edition. CRC Press; 2007.

115. Black DM, Bauer DC, Vittinghoff E, et al. Treatment-related changes in bone mineral density as a surrogate biomarker for fracture risk reduction: meta-regression analyses of individual patient data from multiple randomised controlled trials. The lancet. Diabetes & Endocrinology. 2020 Aug;8(8):672-682. DOI: 10.1016/s2213-8587(20)30159-5. PMID: 32707115.
