# Supplementary material for: Weighted Vest Use or Resistance Exercise to Offset Weight Loss–Associated Bone Loss in Older Adults: A Randomized Clinical Trial
Source: JAMA Netw Open. 2025 Jun 20;8(6):e2516772. doi: 10.1001/jamanetworkopen.2025.16772 (PMC12181796; doi:10.1001/jamanetworkopen.2025.16772)
Supplement: Supplement 3. — Data Sharing Statement [file jamanetwopen-e2516772-s003.pdf]

Data Sharing Statement

Beavers. Weighted Vest Use or Resistance Exercise to Offset Weight Loss–Associated Bone Loss in Older Adults. *JAMA Netw Open*. Published June 20, 2025.
doi:10.1001/jamanetworkopen.2025.16772

Data
Additional Information: ClinicalTrials.gov Registration: NCT04076618
https://clinicaltrials.gov/study/NCT04076618?term=INVEST%20in%20bone%20health&rank=1
Data available: Yes
Data types: Deidentified participant data, Data dictionary
How to access data: kbeavers@wakehealth.edu
When available: beginning date: 05-01-2025

Supporting Documents
Document types: None

Additional Information
Who can access the data: Upon reasonable request and once the request is approved.
Types of analyses: For a specific purpose.
Mechanisms of data availability: With investigator support and after approval.
